# Characterization of *Batrachochytrium dendrobatidis* Inhibiting Bacteria from Amphibian Populations in Costa Rica

**DOI:** 10.3389/fmicb.2017.00290

**Published:** 2017-02-28

**Authors:** Joseph D. Madison, Elizabeth A. Berg, Juan G. Abarca, Steven M. Whitfield, Oxana Gorbatenko, Adrian Pinto, Jacob L. Kerby

**Affiliations:** ^1^Department of Biology, University of South DakotaVermillion, SD, USA; ^2^Centro de Investigación en Estructuras Microscópicas, Universidad de Costa RicaSan Pedro de Montes de Oca, Costa Rica; ^3^Conservation and Research Department, Zoo MiamiMiami, FL, USA; ^4^Life Science Laboratory, Westcore DNA Sequencing Facility, Black Hills State UniversitySpearfish, SD, USA; ^5^Departamento de Bioquímica, Escuela de Medicina, Centro de Investigación en Biología Celular y Molecular, Universidad de Costa RicaSan Pedro de Montes de Oca, Costa Rica

**Keywords:** amphibian, microbiome, *Serratia marcescens*, RNA-sequencing, *Batrachochytrium dendrobatidis*

## Abstract

Global amphibian declines and extinction events are occurring at an unprecedented rate. While several factors are responsible for declines and extinction, the fungal pathogen *Batrachochytrium dendrobatidis* (*Bd*) has been cited as a major constituent in these events. While the effects of this chytrid fungus have been shown to cause broad scale population declines and extinctions, certain individuals and relict populations have shown resistance. This resistance has been attributed in part to the cutaneous bacterial microbiome. Here, we present the first study characterizing anti-*Bd* bacterial isolates from amphibian populations in Costa Rica, including the characterization of two strains of *Serratia marcescens* presenting strong anti-*Bd* activity. Transcriptome sequencing was utilized for delineation of shifts in gene expression of the two previously uncharacterized strains of *S. marcescens* grown in three different treatments comprising *Bd*, heat-killed *Bd*, and a no *Bd* control. These results revealed up- and down-regulation of key genes associated with different metabolic and regulatory pathways. This information will be valuable in continued efforts to develop a bacterial-based approach for amphibian protection as well as providing direction for continued mechanistic inquiries of the bacterial anti-*Bd* response.

## Introduction

The role of the bacterial microbiome in conferring disease resistance has been the subject of intensive study in a number of disease systems. This area has critical importance in elucidating the mechanisms of disease dynamics in the context of important host relationships with bacteria. Inquiries into such systems is an important frontier in many taxa undergoing biodiversity loss. Examples include diseases such as white-nose syndrome caused by *Pseudogymnoascus destructans* in bats (Hoyt et al., 2015) and chytridiomycosis caused by *Batrachochytrium dendrobatidis* (hereafter *Bd*) and *Batrachochytrium salamandrivorans* (hereafter *Bsal*) in amphibians (Longcore et al., 1999; Martel et al., 2013). There are also several anthropocentric reasons to decipher these relationships. Indeed, the role of the bacterial microbiome in agriculturally important plant diseases (Berendsen et al., 2012) and medical applications in human disease (Schwabe and Jobin, 2013; Kostic et al., 2014) necessitates the need for basic research in this field of study. Specifically, the underlying genetic mechanisms that bacteria utilize in mitigating disease risk is an understudied area in disease dynamics. While previous work has examined bacterial gene expression in response to disease in humans (Duran-Pinedo et al., 2014), this is generally lacking in many of the other important disease systems of animals where conservation priorities are a concern.

This study seeks to characterize amphibian cutaneous bacteria and their role in ameliorating amphibian population declines. These declines have been attributed to a variety of factors including habitat loss, global climate change, disease, and environmental contaminants (Collins and Storfer, 2003). In conjunction with other factors, disease has been directly attributed to extinction of various amphibian species (Crawford et al., 2010). Specifically, the *Ranavirus* of the Iridoviridae and the fungal pathogens *Bd* and the now emerging *Bsal* are of concern vis-à-vis the health of at-risk amphibian populations (Martel et al., 2013).

In the wake of devastation that often follows these epizootic events, there are sometimes small relict populations that seem to have an innate resistance to avoid disease (Chaves-Cordero et al., 2014). This survival has been attributed to a variety of factors including the cutaneous bacterial microbiome and host-produced skin peptides (Woodhams et al., 2007a,b,c). An adaptive immune response has also been investigated with results that vary by amphibian species and stage of development (Rollins-Smith, 1998; Ramsey et al., 2010; Poorten et al., 2015). While all of these aspects are important, the possibility of developing probiotic based protection through exploitation and manipulation of the amphibian cutaneous bacterial microbiome has gained recognition as a major research objective. It has been shown that while whole bacterial community composition is important (Rebollar et al., 2016a), there are often specific bacterial species with strong anti-*Bd* properties (Harris et al., 2009). While previous work has examined the transcriptomic response of Anurans to *Bd* (Rosenblum et al., 2012a; Ellison et al., 2014; Price et al., 2015) and also the transcriptome of *Bd* (Rosenblum et al., 2008, 2012b), there have been no studies delineating the transcriptomic response of bacteria to *Bd*. However, there have been studies examining the bacterial metabolites produced in known anti-*Bd* bacteria (Brucker et al., 2008a,b; Belden et al., 2014; Loudon et al., 2014). One important bacteria that has been the subject of various papers is the anti-*Bd* bacteria *Janthinobacterium lividum* (Brucker et al., 2008a). This bacteria produces a secondary metabolite, violacein, which has important similarities to the secondary metabolite prodigiosin produced in another known anti-*Bd* bacteria, *Serratia marcescens*. *J lividum* is also known to produce extracellular chitinases similar to those of *S. marcescens* (Gleave et al., 1995). These similarities could present an important pathway for general anti-*Bd* inhbition. These studies also provide an important overall context for interpreting results presented herein. The need for work addressing these issues has been suggested in recent communications (Woodhams et al., 2016).

To explore the mechanisms by which individual bacteria may be deterring *Bd* growth and thus allowing amphibian persistence in a disease outbreak, our group has sampled various relict, recovering, and unaffected amphibian populations in Costa Rica. Bacterial species were isolated in pure culture and assayed for *Bd* inhibition. Of those bacteria that were determined to be strong anti-*Bd* candidates, two strains of *S. marcescens* were selected for further characterization. Previous studies have examined other *Serratia* spp. that have known anti-*Bd* activity *in vitro* (Woodhams et al., 2014; Becker et al., 2015). It was hypothesized that there would be significant gene up- and down-regulation in *S. marcescens* gene expression as a response to *Bd* which could highlight transcriptomic shifts associated with specific bacterial response mechanisms. Also examined was the expression of genes that are involved in canonical *S. marcescens* antifungal pathways. Known antifungal pathways include the production of broadly antifungal enzymes such as extracellular chitinases and glucanases, as well as the production of secondary metabolites such as prodigiosin (Duzhak et al., 2012; Gutiérrez-Román et al., 2015; Tan et al., 2015). Elucidations from this data will allow for more in-depth studies to occur on the mechanisms by which *S. marcescens* inhibits *Bd* growth. Such mechanistic determinations could be utilized in work developing probiotic bioaugmentation tools for use on both captive and wild amphibian populations.

## Materials and methods

### Bacterial isolation

**Figure 1:**
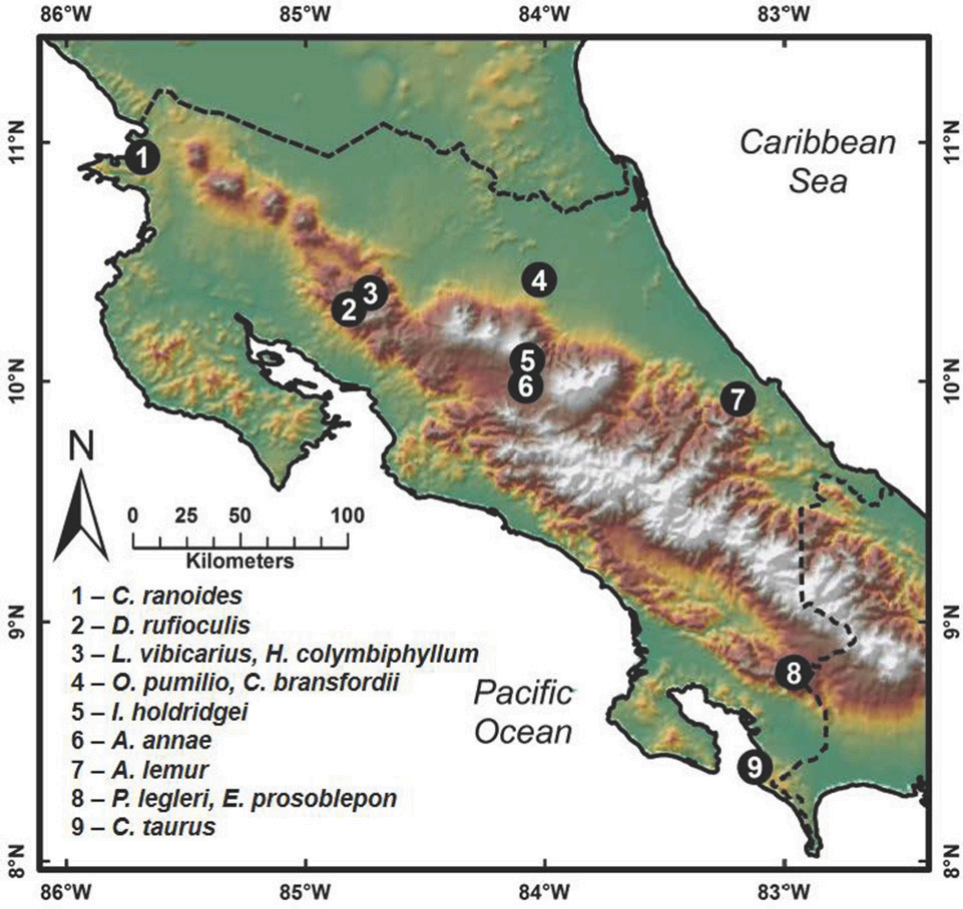
**Map showing amphibian collection sites in Costa Rica where amphibians were swabbed for bacterial isolates**. Species collected at each site are given.

Field sampling for cutaneous bacteria was conducted between March and November 2012 by searching for amphibians in relict populations at sites where they had been previously reported (Puschendorf-Fahrenkrug et al., 2005; Hoffmann, 2006; Abarca et al., 2010; Chaves-Cordero et al., 2014). Bacterial isolates were obtained by swabbing 191 frogs belonging to 12 amphibian species in neotropical montane regions of Costa Rica (Figure 1; species collected given in Supplementary Table 4). Frogs were captured with plastic bags and handled using fresh disposable nitrile gloves. The capture of transient surface bacteria was reduced by washing the entire body of individuals for seven seconds with sterile distilled water. Individuals were swabbed over the entire body using a sterile cotton swab. First, swabs were streaked onto the surface of Reasoner's 2A (R2A) agar in a petri dish to obtain bacteria; subsequently the washing was continued with sterile distilled water, and a second swab from the frog was streaked onto chitin agar media for obtaining actinomycetes. Plates were cultured at 25–28°C and colony formation was observed daily. In the case of R2A media, the formation of bacterial colonies was observed at 2 or 3 days of isolation, and one to 2 weeks for actinomycetes. The different colonies were then purified on a new medium: Luria-Bertani (LB) for bacteria from R2A media and yeast malt extract agar (YMEA) for actinomycetes. Each bacterial isolate was cryopreserved with liquid LB media with 40% glycerol and YMEA liquid media with 20% glycerol, respectively. All work with amphibians was carried out in accordance with the recommendations of the Institutional Commission on Biodiversity at the University of Costa Rica. Amphibian sampling resulting in the bacteria used in this study was done in conjunction with Adrian Pinto at the University of Costa Rica, with research permits approved by the Institutional Commission on Biodiversity at the University of Costa Rica (Resolutions 014).

### Cell-free supernatant challenge assay

A cell-free supernatant assay was initially used to examine anti-*Bd* potential of bacterial isolate extracts. This assay followed closely to the protocol outlined by Bell et al. (2013). Briefly, bacterial isolates were first inoculated into 2 mL of sterile tryptone gelatin hydrolysate lactose (TGhL) medium and incubated at 25°C for 48 h. They were then centrifuged at 5,796 g for 10 min to pellet cells. The supernatant was transferred to 1.5 mL filter microtubes and centrifuged again at 5,796 g for 10 min until only peptides remained suspended in the broth. Experimental and control wells were divided on 96-well microplates. The experimental wells contained 50 μL of *Bd*-inoculated TGhL broth (3.15 × 106 zoospores per mL), 45 μL TGhL medium, and 5 μL of the bacterial isolate. Three controls were used. The positive control wells contained 50 μL of *Bd*, 45 μL TGhL medium, and 5 μL deionized water. The negative control wells contained 50 μL of heat killed *Bd* (raised to 60°C for 30–60 min), 45 μL of TGhL medium, and 5 μL deionized water. The final wells were blanks and contained 95 μL TGhL medium and 5 μL deionized water. Complete plates contained four replicates of each bacterial isolate and eight replicates of the positive, negative, and medium-only controls. Absorbance values were recorded after plate setup was completed (day 0) and then every 24 h on a spectrophotometer (BioTek Take 3) at 492 nm until maximum *Bd* growth (day 14). Plates were kept at room temperature in the dark between absorbance value recordings. Ninety bacterial isolates were examined using this method. The mean daily absorbance values of all replicate and control wells were calculated daily using a spectrophotometer. A generalized linear model (GLM) was used to compare each isolate with replicated controls. Increasing absorbance indicates zoospore reproduction and growth, while decreasing absorbance indicates zoospore death.

### Agar plate challenge assay

For the agar plate challenge assay, plates with TGhL agar were inoculated with a lawn of *Bd* (strain JEL 731, isolated from *Craugastor bransfordii* at La Selva Biological Station, Costa Rica). This lawn was then streaked with one of the bacterial isolates with each isolate being examined in triplicate. Each bacteria was incubated for 48–72 h at 25°C and then flooded with 3 mL of *Bd*-inoculated broth (3.15 × 10^6^ zoospores per mL) and left for 1–2 h until agar had absorbed most of the *Bd*-inoculated broth. Each plate had two bacterial strains, with pairs randomized across four replicates. Pictures were taken of the bacteria every 24 h for 3 days. ImageJ software was used to standardize measurements. Zone of inhibition (ZOI) was measured by calculating the area of each zone [Area = (ZOI length–bacteria length) × (ZOI width–bacteria width)]. A GLM was used to compare each isolate to a theoretical control where the ZOI = 0. All of the isolates but one exhibited a ZOI, but only 38 of the 86 had a significant *p*-value (*p* < 0.05). After 2 days of growth at 20°C, the resulting zone of inhibition was quantified. Of those bacteria that were determined to be strong anti-*Bd* candidates and identified using 16S rDNA sequencing, two strains of *S. marcescens* were selected for further characterization. The two strains had 91% 16S rDNA partial sequence homology (BLAST Needleman-Wunsch alignment). Both strains of S. marcescens used in this study were isolated from a single captured and released specimen of *Agalychnis annae* from a successfully translocated population established from a relict population in Costa Rica.

### 16S rDNA sequencing

DNA was extracted from 36 bacterial isolates with the greatest *Bd-*inhibitory properties. DNA extraction utilized a Qiagen DNeasy Blood and Tissue kit (Qiagen-Hilden, Germany). DNA preparation for sequencing was done according to manufacturer directions. Briefly, extracted DNA was amplified using the eubacterial primers 27f (5′-AGAGTTTGATCMTGGCTCAG-3′) and 1492r (5′-ACCTTGTTACGACTT-3′). This amplified product was then sequenced with the original primers as well as the internal primers 907r (5′-CCGTCAATTCMTTTRAGTTT-3′) and 704f (5′-GTAGCGGTGAAATGCGTAGA-3′). The BigDye Terminator v3.1 cycle sequencing kit was utilized in sequencing. Sequencing was carried out using an Applied Biosystems 3500 Genetic Analyzer (Life Technologies-Carlsbad, California, USA). The output sequencing data was assembled with Geneious (version 7.0) and subjected to taxonomic identification using BLAST in the NCBI database.

### Bacterial and fungal growth analysis

All fungal growth was done in lactose TGhL broth as per previous studies (Bell et al., 2013). Growth curves of *S. marcescens* were also acquired under the varying treatments (control, Bd, heat-killed Bd). These growth curves were acquired using serial dilutions followed by plating and counting of colony forming units to avoid the non-distinguishing reading of zoospores from bacterial cells by the spectrophotometer which would have resulted in skewed bacterial cell counts between treatments.

### RNA-sequencing

**Figure 2:**
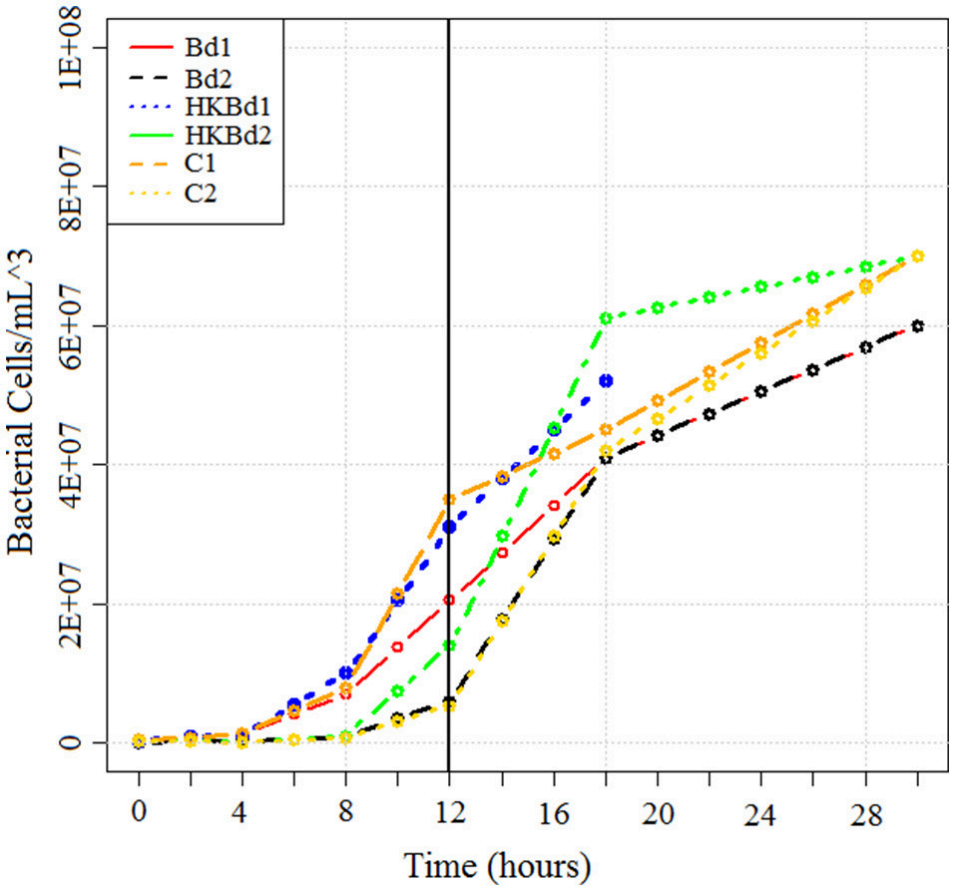
**Growth curve of bacteria in all treatments obtained using serial dilution plating**. RNA was extracted at *T* = 12 h (indicated by vertical line). Bd1 and Bd2 indicate *S. marcescens* strains one and two grown with *Bd*, respectively. Heat-killed Bd1 and heat-killed Bd2 indicates *S. marcescens* strains one and two grown with heat killed *Bd*, respectively. No Bd1 and no Bd2 indicates *S. marcescens* strains one and two grown without any *Bd*, respectively.

*Bd* strain JEL731 was used in this experiment. *S. marcescens* (described above) were isolated from the cutaneous layer of *A. annae*. Both *S. marcescens* strains were subsequently shipped from Costa Rica to the University of South Dakota and grown in culture on LB agar. TGhL broth was inoculated with *Bd*, mixed, and spiked with *S*. *marcescens* according to our experimental conditions. Each experimental condition was done in triplicate with bacterial populations of both strains (at 20°C). Experimental conditions included a no-*Bd* zoospore control, heat-killed *Bd* zoospore control (killed by exposure to 20 min at 50°C; Johnson et al., 2003), and live-*Bd* zoospores. The concentration of *Bd* zoospores used for both heat-killed and live-*Bd* zoospore experimental conditions were 25 × 10^4^ zoospores/mL (measured with a hemocytometer). Cultures were incubated at 20°C with RNA being extracted in bacterial exponential growth phase at 12 h (Figure 2). RNA extraction was done by initially stabilizing bacterial RNA with RNAprotect bacteria reagent (Qiagen-Hilden, Germany). Enzymatic lysis was subsequently used using an EDTA (ethylenediaminetetraacetic acid)/lysozyme solution buffered with Tris. RNA purification was then carried out using an RNeasy kit (Qiagen-Hilden, Germany) following standard protocols. Purified RNA was then transported from the University of South Dakota to the WestCore DNA core facility for sequencing preparation and sequencing. Subsequent rRNA depletion of total-RNA for both bacteria and fungi was accomplished using a modified protocol for a Ribo-Zero rRNA Removal Kit (Illumina, San Diego, CA). The modified protocol included probes from both the yeast and bacterial rRNA Removal Kits which were combined in a 1:1 ratio to the recommended concentration for a one probe kit. Clean-up of the rRNA reduced RNA was done using a RNA-Clean and Concentrator kit (Zymo Research-Irvine, CA) following manufacturer instructions. RNA quality (RIN score) was obtained on a LabChip GX (Caliper, a PerkinElmer company, Hopkin, MA) using the PicoRNA assay. RNA quantity was measured on a Qubit 2.0 Fluorometer (Thermo Fisher Scientific, Waltham, MA, USA). Subsequent library preparation for sequencing utilized the Illumina ScriptSeq RNA-Seq Library Kit. Library quantification was done with a LabChip GX using the DNA High Sensitivity assay and Qubit 2.0 Fluorometer. Indices were added for a six sample multiplex on a one lane flowcell. The Illumina MiSeq platform was used for the sequencing run using the version 3 reagents kit to obtain 2 × 76 bp reads (paired-end).

### qPCR verification

A subset of differentially expressed genes (DEGs) identified through RNA-sequencing were verified using RT-qPCR. RNA was from the same samples utilized in the RNA sequencing and were reverse transcribed to cDNA using the Quantitect Reverse Transcription Kit (Qiagen-Hilden, Germany). Real-time PCR was done on the StepOnePlus Real-Time qPCR-system (Applied Biosystems-Foster City, California) using SYBR Green chemistry as provided by the SYBR Green PCR Kit (Qiagen-Hilden, Germany). All qPCR was carried out with no-RT and no-template negative controls. The *narG* (nitrate reductase) gene and *fadE* (acyl-conezyme A dehydrogenase) gene were examined due to their significant upregulation observed in RNA-seq analysis. Other genes examined included *chiA* (chitinase A) and *pigM* (key-regulatory enzyme in the prodigiosin production pathway) to confirm lack of differential expression as seen in RNA-seq analysis. The reference genes *dnaE* and *rplU* were utilized for statistical comparison. Every gene analyzed had expression determined under every experimental condition (*Bd* treatment, HK-*Bd* treatment, and no-*Bd* control). All primer sequences used were either from the literature or designed in NCBI using the Primer BLAST tool followed by verification in our laboratory (primer sequences, Supplementary Table 1).

### Statistical analyses

De-multiplexing of raw data was done on Illumina BaseSpace as part of the BaseSpace MiSeq Reporter workflow. Analysis of raw RNA-seq data was done using the Rockhopper 2 software platform (McClure et al., 2013; Tjaden, 2015). Raw g-zipped fastq files were uploaded directly to the Rockhopper platform with paired-end reads being combined. Assembly and mapping of the raw data was done using the default settings. Reads were mapped to the *S. marcescens* WW4 reference genome (NCBI Reference Sequence NC_020211.1). Significant differential gene expression analysis was also done using Rockhopper 2. Briefly, assembled/mapped files were subjected to upper-quartile normalization. Expression data was then subjected to the Rockhopper algorithm assuming a negative binomial distribution for estimation of *p*-values. *P*-values were then used to obtain q-values in which false discovery rate is taken into account using the Benjamini-Hochberg procedure. Analysis of qPCR data for differential gene expression was completed using the ΔΔCt method (Livak and Schmittgen, 2001).

## Results

### Hosts and isolates

Amphibian species *A. annae, Agalychnis lemur, C. bransfordii*, and *Oophaga pumilio* had the greatest number of anti-*Bd* bacterial isolates (Supplementary Table 3). *A. lemur* hosted two bacterial species that were shown to enhance *Bd* growth. None of the tested isolates from species *Craugastor ranoides, Craugastor taurus*, or *Hyalinobatrachium colymbiphyllum* exhibited anti-*Bd* properties. However, we note that not all of the strains isolated were tested, and bacterial strains are known to exist from *H. colymbiphyllum* that are not culturable or have culturing bias (Walke et al., 2011). *A. annae* exhibited the highest diversity of anti-*Bd* bacteria, with 7 different genera represented in anti-*Bd* isolates.

Identified anti-*Bd* bacteria were in the phyla Proteobacteria, Bacteroidetes, Actinobacteria, and Firmicutes. Proteobacteria was the most common phyla being found on 5 of the 12 amphibian species. The following genera were also identified: *Alcaligenes, Bacillus, Chyrseobacterium, Enterobacteriaceae, Lysinibacillus, Microbacterium, Pseudomonas, Sphingobacterium, Staphylococcus*, and *Stenotrophomonas*. The *Serratia* genus was the most common identified within the selected anti-*Bd* bacteria isolates; *S. marcescens* was the most common species (Supplementary Table 5) and was found on three of the amphibian species.

### Bacterial challenge assays

**Figure 3:**
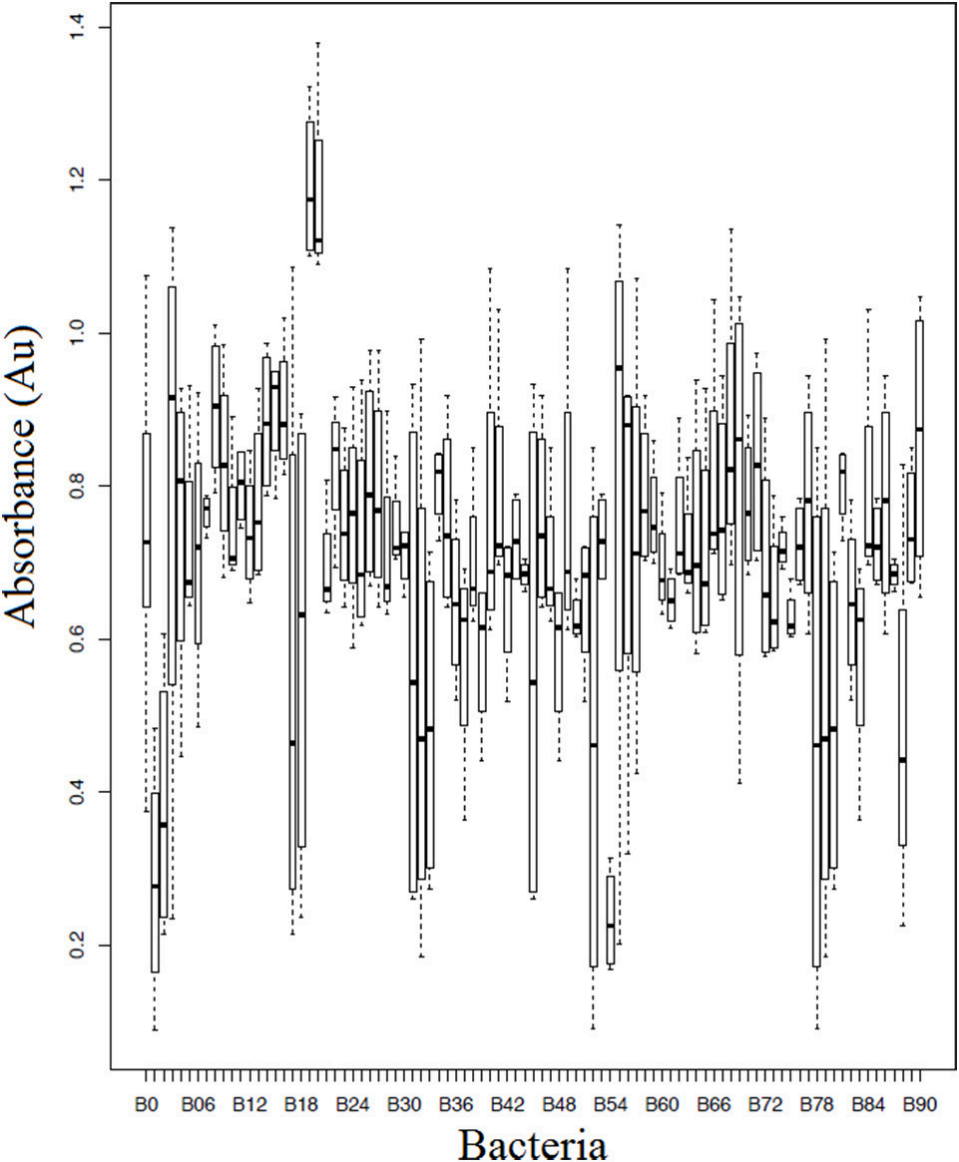
**Box plot estimates of the cell-free supernatant challenge assay for each of the bacteria strains**. The box represents the interquartile range (IQR) and the top and bottom whiskers represent Q3—(1.5 IQR) and Q1—(1.5 IQR), respectively. Bacteria IDs on the x-axis are referenced in Supplementary Table 3. Lower values signify greater inhibition of *Bd* growth. B0 corresponds to a *Bd*-only control.

**Figure 4:**
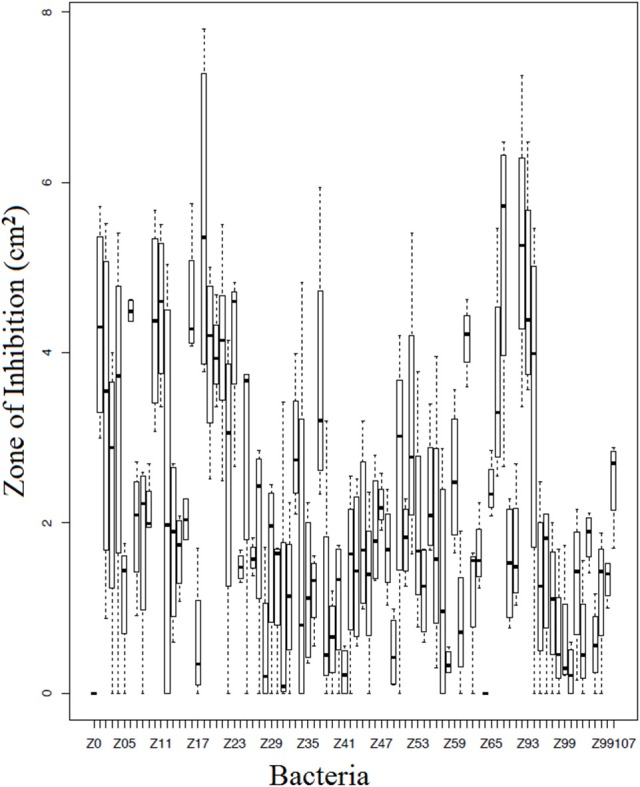
**Box plot estimates of the agar based challenge assay for each of the bacteria strains**. The box represents the interquartile range (IQR) and the top and bottom whiskers represent Q3—(1.5 IQR) and Q1—(1.5 IQR), respectively. Bacteria IDs on the x-axis are referenced in Supplementary Table 4. Larger values signify greater inhibition of *Bd* growth.

Of the 90 bacterial isolates examined in the cell-free supernatant challenge assay, 10 (11.1%) exhibited *Bd* inhibition where *p* < 0.05 and 2 (2.2%) enhanced *Bd* growth (Figure 3; Supplementary Table 2). Of the 86 isolates examined in the agar-based challenge assay (Figure 4; Supplementary Table 3), 38 (44.2%) of isolates were characterized as anti-*Bd* (*p* < 0.05). All but one isolate produced a zone-of-inhibition during the experiment (Supplementary Table 3).

We also compared the 68 isolates which were examined in both assays; overall 30 exhibited anti-*Bd* properties (44.1%; Supplementary Table 4). Of these 30 isolates, 28 isolates (93.3%) were shown to inhibit *Bd* using the agar-based method but not the cell-free supernatant method, and four isolates (13.3%) were shown to inhibit *Bd* using both methods. There were 2 isolates that exhibited anti-*Bd* properties (*p* < 0.05) in the cell-free supernatant assay but not in the agar-based assay.

### Transcriptomic analysis

**Figure 5:**
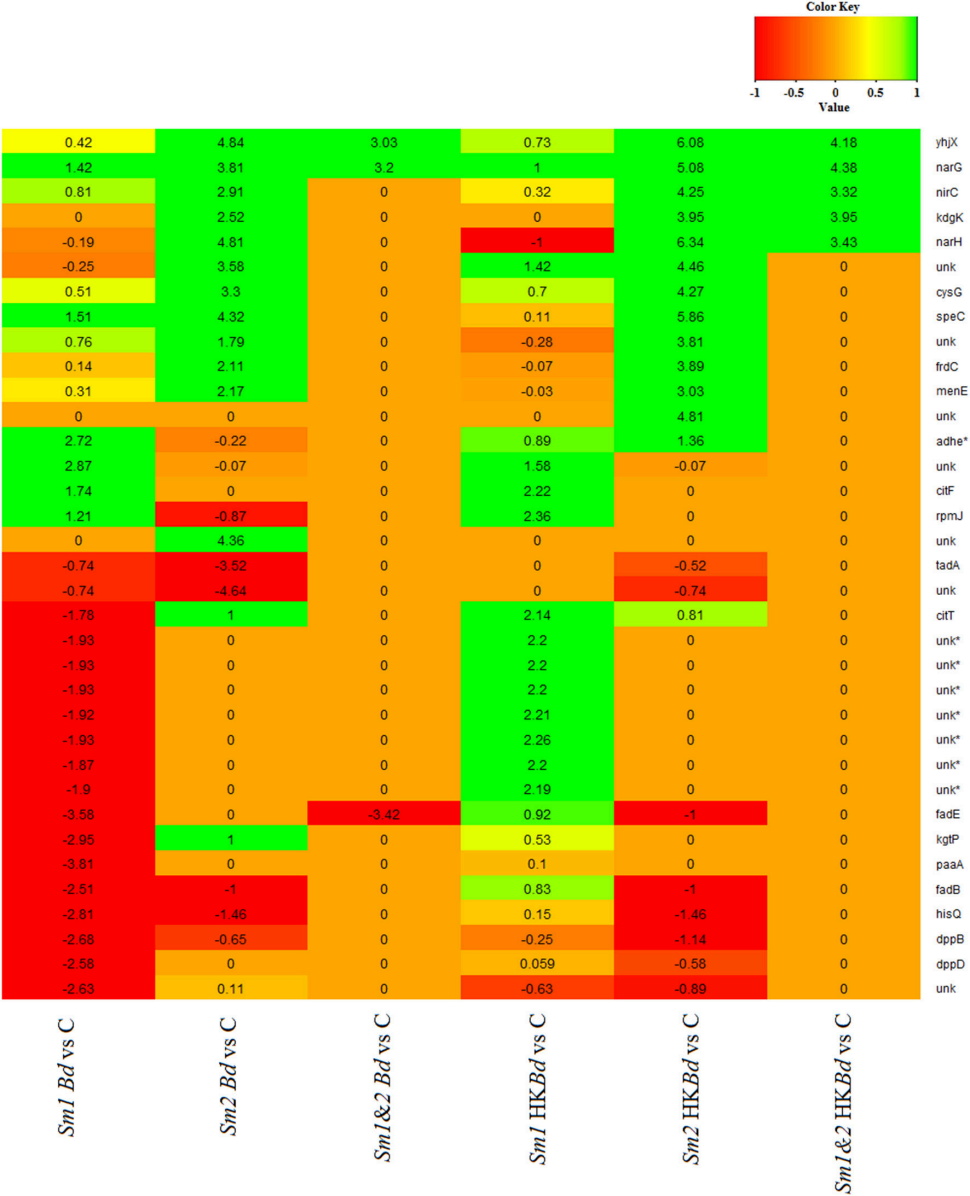
**Gene expression data presented as a heat map for the 15 most up- and down-regulated genes for *Bd* and heat-killed *Bd* treatments relative to a no-*Bd* control**. Corresponding gene expression values are given for those only in the top 15 in one treatment. Corresponding genes that were not significantly up or down-regulated relative to a no-*Bd* control but were in the top 15 in a different treatment are denoted by a value of 0. Gene names are given on the vertical axis. Treatments are given on the bottom horizontal axis. Sm1 and Sm2 denote *S. marcescens* strain one and two. Bd indicates live *B. dendrobatidis* and HKBd indicates heat-killed *B. dendrobatidis*. 1.2 indicates pooled expression data for both strains. A gene name of *unk* indicates an unnamed gene. An asterisk (^*^) by a gene name indicates an antisense gene.

**Figure 6:**
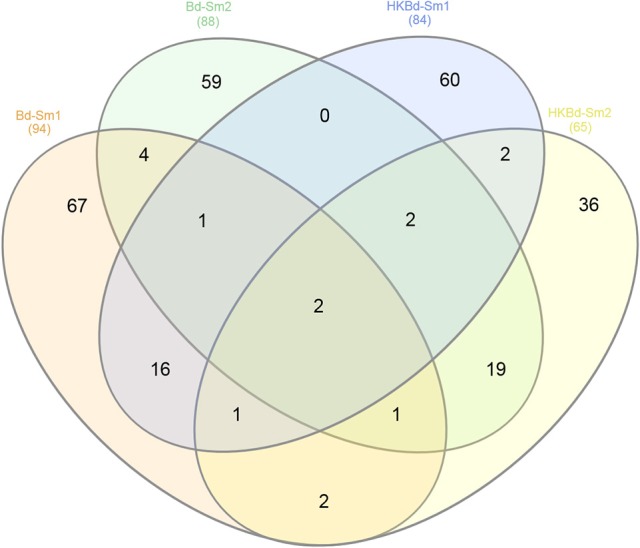
**Venn diagram depicting shared significantly expressed DEGs from each of the treatment conditions (*Bd* and heat killed-*Bd*; both vs. no-*Bd* control) in both *S*. *marcescens* isolates examined**.

All extracted RNA had RNA integrity numbers (RINs) >9.0 indicating that all extracted RNA samples were within tolerance for use in sequencing. Raw sequencing data of two *S. marcescens* strains from *A. annae* were referenced against the *S. marcescens* WW4 genome with >80% mapping (Table 1). Additionally, 47–74% of reads were mapped to protein coding genes (Table 1). All DEGs identified in each treatment were compared to a no-*Bd* control. In the live *Bd* treatment this experiment found 111 differentially expressed genes (DEGs) in *S. marcescens* strain one (Supplementary Table 6) and 100 DEGs in *S. marcescens* strain two (Supplementary Table 7). For the heat-killed *Bd* treatment, *S. marcescens* strain one had 96 DEGs (Supplementary Table 8) and *S. marcescens* strain two had 73 DEGs (Supplementary Table 9). Pooling results from both strains of *S. marcescens* used in this study, only three significant DEGs were identified in the *Bd* treatment while five significant DEGs were identified in the heat-killed *Bd* treatment. We were able to identify the top 15 up- and down-regulated genes in each treatment in each strain as well as the significant DEGs that were shared with both strains pooled (Figure 5). Genes that were significantly upregulated in both strains included those associated with the nitrate reductases and the associated transport machinery (*narG, narH*, and *nirC*). Also of interest and shared between both strains was a putatative oxalate-formate antiporter coding gene (*yhjX*) and the acyl-conezyme A dehydrogenase gene (*fadE*). We also used a Venn diagram to visualize the differentially expressed genes of *S. marcescens* in strains one and two as a response to live-*Bd* and heat-killed *Bd* (Figure 6).

**Table 1:** **Sequencing results including total reads for each multiplexed sample, reads mapped to reference genome, and mapped genes aligning to protein coding genes**.

^*^Sequenced sample	**Total reads**	**Mapped reads**	**Aligning to protein coding genes**
SMWW4 Control (1)	3239079	2664279 (82%)	1491996 (56%)
SMWW4 Control (2)	3814486	3258485 (85%)	2378694 (73%)
SMWW4 *Bd* (1)	3938104	3201797 (81%)	2145204 (67%)
SMWW4 *Bd* (2)	3912551	3348231 (86%)	2477691 (74%)
SMWW4 HK*Bd* (1)	4097349	3361055 (82%)	1579696 (47%)
SMWW4 HK*Bd* (2)	3139545	2623075 (84%)	1914845 (73%)

* *Both strains were mapped to the S.marcescens strain WW4 (SMWW4) with correlating experimental growth condition (Control, B. dendrobatidis present (Bd), heat-killed B. dendrobatidis present (HKBd) and biological replicate number*.

**Figure 7:**
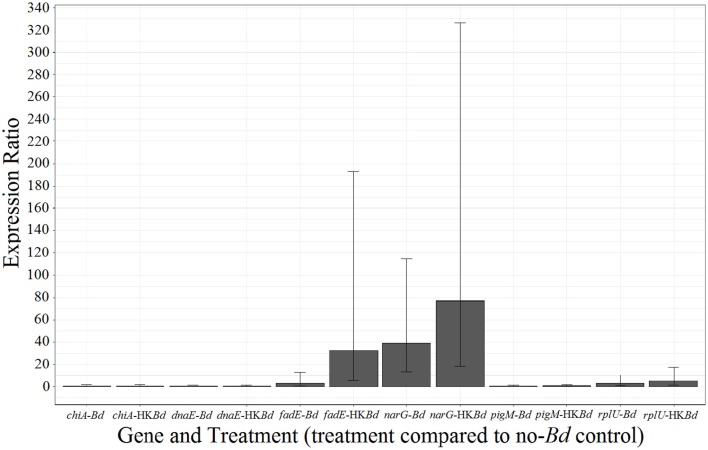
**qPCR verification of a select group of genes identified in RNA-sequencing**. Error bars represent 95% confidence intervals. All gene expression was compared to *dnaE* and *rplU* reference genes.

RNA-sequencing expression results were validated by examination of six genes in the presence of the two experimental treatments and control (Figure 7). The *narG* gene showed upregulation in *Bd* and heat-killed *Bd* treatments with much stronger upregulation in response to the live *Bd* treatment. The *fadE* gene was also significantly upregulated but only in the *Bd* treatment. Other genes examined included *chiA* (chitinase A) and *pigM* (key-regulatory enzyme in the prodigiosin production pathway) to confirm the lack of differential expression as seen in RNA-sequencing. Reference genes *dnaE* and *rplU* showed no significant differential expression.

## Discussion

This work is the first to highlight gene expression in anti-*Bd* bacterial strains. More importantly, many of these bacteria were isolated from amphibian species in recovering and relict populations from a major epizootic event. These strains represent important data for understanding the role the bacterial microbiome has in conferring *Bd* resistance. Understanding which bacterial species are anti-*Bd* and the underlying gene expression is meaningful for proposing mechanisms of anti-*Bd* action and subsequent verification studies using gene knockout and complementation. Such work will be invaluable to understanding disease resistance and the role of the bacterial microbiome. Additionally, this work is critical for the future development of novel *in situ* microbiome engineering tools for conservation.

In general, the bacterial genera that were identified in this study as having anti-*Bd* activity were similar to those found in other studies both in the tropics and in temperate zones (Lam et al., 2010; Flechas et al., 2012). The bacterial isolates most common in this study were members of the genus *Serratia*, part of the phylum Proteobacteria. *Serratia* were the strongest candidates for bioaugmentation efforts identified in this study owing to their rapid growth *in vitro*, apparent anti*-Bd* properties**, and common distribution across various Costa Rican amphibian species. They have also been shown to exhibit anti-*Bd* properties in previous work examining amphibian microbiomes (Woodhams et al., 2007b; Antwis et al., 2015). While the *S. marcescens* used in this study have excellent potential for bioaugmentation efforts and mechanistic inquiries, certain strains of *S. marcescens* are known opportunistic human pathogens (Hejazi and Falkiner, 1997). Caution should be taken in working with uncharacterized wild-type strains of this bacteria including safety education of personnel and BSL-2 standards in all laboratory work.

In understanding *S. marcescens* and their anti-*Bd* mechanisms in more detail as it pertains to the cutaneous microbiome, we found that the two *S. marcescens* strains examined in this study had various transcriptomic shifts in response to *Bd*. The responses seen were varied and implicated various metabolic and regulatory processes. The genes validated using RT-qPCR had complete congruency with the RNA-sequencing data. This should be seen as a small but important validation of the significantly expressed genes identified in the RNA-sequencing portion of the study as well as validation of lack of expression in genes coding for enzymes known to have antifungal activity.

The upregulation of nitrate reductases and nitrite transporters was one of the few gene sets shown to be upregulated in both strains and in both treatments. Specifically, the significant upregulation of genes encoding the catalytic nitrate reductase α-subunit and transitory β-subunit which are both part of the membrane bound reductase (NAR) complex is of interest. This data coupled with upregulation of the inner membrane nitrite transporter in both strains seems to indicate a response to anaerobic conditions in which nitrate reduction is utilized for energy conservation (Richardson et al., 2001). The reason for the utilization of the membrane-bound nitrate reductase machinery other than a response to anaerobic conditions and available nitrate is unclear but certainly occurring in both *Bd* and heat-killed *Bd* treatments.

Also upregulated in both strains under both conditions was a putative oxalate-formate antiporter coding gene. The need to upregulate the antiport machinery for oxalate decarboxylation to formate in response to *Bd* is at this point not fully understood. However, the production of oxalate by other fungi and the necessary genes in the *Bd* genome for production of oxalate suggest such production by *Bd* is theoretically possible and potentially necessary (Benny, 1995; Bindschedler et al., 2016). This coupled with recent work showing amphibian mortality due to oxalate nephropathy from an unknown oxalate source may suggest a specific mechanism by which *Bd* could kill amphibians (Tokiwa et al., 2015). This possible mechanism deserves further consideration, especially owing to the fact that specific mechanisms for *Bd* induced mortality are not fully understood. Evidence for the epidermal dysfunction hypothesis presented by Voyles et al. (2009) still has no clear biochemical mechanism delineated for the disruption of epidermal channels involved in electrolyte transport. Whether this response and the formation of nitrite into the periplasmic space has any direct inhibitory effects on *Bd* growth is also unclear but worth further consideration.

Differential expression of the commonly associated antimicrobial genes of *S. marcescens* was not seen. These include the upregulation of extracellular chitinases and glucanases as well as the key regulatory enzymes involved in prodigiosin biosynthesis. Chitinases have been previously shown to target the β-1,4 n-acetylglucosamine linkages of the fungal chitin polymer which constitutes a substantial component of the fungal cell wall (Chet and Inbar, 1994). Also, bacterial glucanases have been shown to have a similar effect in the targeting of glucan linkages critical for fungal cell wall structure (Hong and Meng, 2003). The importance of prodigiosin, a secondary metabolite shown to have antifungal characteristics, was also examined in the context of the key regulatory enzymes of the prodigiosin biosynthesis pathway. These enzymes, which are encoded by the prodigiosin biosysnthesis gene cluster (*pig* cluster), were not shown to be upregulated in this study. The downstream effects of this lack of expression was also confirmed visually as *S. marcescens* in culture exhibited only a faint red color (color consistent with the production of the red pigment prodigiosin) under the experimental conditions used in this study. This can most likely be attributed to the known temperature dependence of prodigiosin production in *S*. spp. (Williams, 1973; Woodhams et al., 2014). The temperatures used for co-culturing in this study (20°C) are not generally known to be the most advantageous for prodigiosin production. The lack of upregulation seen in the chitinase (*chi*) genes as well as those involved in glucanase production was also of interest. While not being upregulated directly, there was still a consistent amount of basal expression which may indicate constitutive production of these products which is unaffected by the presence of *Bd* or heat-killed *Bd*. While differential expression is of interest and often informative of an organism's response (e.g., bacteria to *Bd*) there may be products that are basally expressed and indifferent to the presence of a stimulatory organism such as *Bd*. The only way in which to empirically evaluate the importance of such products being produced in an anti-*Bd* response would be the generation of knockouts or knockdowns of the genes of interest in line with molecular Koch's postulates (Falkow, 1988).

While not showing differential expression, the observed expression of the proteins associated with the type six secretion system (T6SS) was also of interest. The expression of a majority of the 13 core genes including *VgrG* (*TssI*) and *Hcp* (*TssD*) associated with the T6SS could have implications in direct inhibition of *Bd* by *S. marcescens*. While the use of such a system has been shown to inhibit competing bacteria in other *S. marcescens* strains (Murdoch et al., 2011), the existence of effector proteins associated with the T6SS that are antifungal in nature are at this point unclear. However, direct competition with other bacteria could allow *S. marcescens* to propagate to higher levels at the bacterial community level allowing other factors discussed above to play a larger role in the system. This preliminary evidence on the role of a T6SS in *S. marcescens* and other gram negative bacteria in the amphibian-*Bd* system therefore merits further examination.

The upregulation of 2-dehydro, 3-deoxy-phosphogluconate aldolase gene, coding for a key regulatory enzyme in the Entner-Duodoroff (ED) pathway, was seen in both *S. marcescens* strains in response to heat-killed *Bd*. The reason behind this upregulation in response to both strains of heat-killed *Bd* treatments is unclear but could also merit further consideration.

The upregulation of various antisense-RNAs was also intriguing and could represent a point of regulation which is part of a response to *Bd*. All asRNAs were associated with ribosomal RNAs representing possible regulation of translation. Curiously, all differentially expressed asRNAs were only observed in strain one with down-regulation as a response to *Bd* and upregulation as a response to heat-killed *Bd*. These results seem to indicate clear differences of a bacterial reaction to *Bd* and heat-killed *Bd* in strain one. The generation of asRNAs are known to have significant regulatory effects on gene expression as well as functional roles (Sesto et al., 2013). Specifically, asRNAs have been shown to induce (i.e., *agr* locus of *Staphylococcus aureus*) as well as attenuate (i.e., asRNA dependent *rep*-mRNA conformations for attenuation of replication control) various bacterial functions (Brantl, 2002). However, the exact role of such regulation and function in this system remains unclear.

The two strains of *S. marcescens* used in this study both had high homology to sequenced strain WW4. However, there are obvious physiological differences in these strains as indicated by the few (eight) genes that were differentially expressed in both. The strains used were collected at the same time but remained in culture for different amounts of time before being cryoarchived representing a possible source of the observed differences. The unstable genomes of wild-type bacteria and potential for lateral gene transfer could account for differences in the aforementioned timeframe and highlights the difficulty in working with such bacteria. While the inclusion of more biological replicates could have been beneficial, both strains came from the same individual and were the only two strains collected in suburban Heredia, Costa Rica. It should also be noted that each sample of assumedly clonal bacterial cells should have variation following an approximate normal distribution due to the high population (*n* > 10,000,000) of bacterial cells in each tube. Setting up multiple growth flasks would be a better indicator of population sensitivity (and resulting expression profile) to small and potentially uncontrollable differences in growth conditions. We have included results from both the comparisons between individual bacterial populations as well as comparisons with both populations pooled. The introduction of other *S. marcescens* from other amphibian species and individuals would nullify the comparison of bacteria growing in the same environment although would still be interesting but ultimately outside the scope of this study.

The results from the transcriptomic analysis portion of this study provide important information of a bacterial species with strongly inhibitory *Bd* properties. The use of meta-omics approaches as suggested by Rebollar et al. (2016b) could also be utilized in further studies delineating whole bacterial microbiome response to *Bd*. However, we suggest that conclusions delineating species-specific bacterial mechanisms of interest for probiotic development from such methods should be done with caution. Many bacteria identified through such techniques have no reference genome or at the least have large repeat regions creating challenging areas for *de novo* assembly in species-specific determinations (NP-hard). Interpreting data and trusting available workflows is also problematic and fails to recognize these considerations in such analyses. Determination of error in workflows can also be challenging and would entail complex error propagation (e.g., Taylor series expansion) strategies that are currently limited in implementation. Any meta-omic interpretations should be reserved for the community-level in the interest of excluding erroneous conclusions. Such data would still be of great benefit in continued research initiatives.

Several of the amphibian species examined had few to no anti-*Bd* bacterial isolates. The number of isolates examined from each amphibian species is small compared to the breadth of the entire microbial community on the cutaneous layer. Additionally, not all isolates from field sampling were used in this study. Microbial communities on amphibians are extremely variable between populations and individuals within the same population across temporal and spatial distributions (Vredenburg et al., 2010). Continued sampling and bioassays of the bacteria of these relict populations may uncover additional bacteria that exhibit anti-*Bd* properties. Additionally, the number of anti-*Bd* isolates does not necessarily correlate with the success of an amphibian population; few isolates may still provide protection against chytridiomycosis.

It is also possible that these relict populations have not yet acquired anti-*Bd* bacteria, but instead persist in the presence of *Bd* because of other immune defenses. While symbiotic bacteria are an effective defense against *Bd*, other innate defenses such as antimicrobial peptides (AMPs) secreted by dermal glands are important in conferring anti-*Bd* properties (Rollins-Smith and Conlon, 2005; Conlon, 2011). AMP secretion has been shown to be variable between amphibian species and families creating many possible dynamics between AMPs and the amphibian bacterial microbiome (Conlon, 2011). Three of the amphibian species examined in this study belong to families known to secrete AMPs with anti-fungal properties and also host isolates identified as anti-*Bd* by this study (Apponyi et al., 2004; Amiche et al., 2008; Conlon, 2011). However, *Incilius holdridgei* of the Bufonidae family are known to not secrete any distinguishable AMPs with antifungal properties. Interestingly, this species also lives in areas with the ideal environmental conditions for *Bd* growth (Abarca et al., 2010) thus making it possible that symbiotic bacteria play a strong role in resistance to chytridiomycosis. In addition to innate immune responses, the adaptive immune system can also provide significant benefit in anti-*Bd* activity. The relationship between innate and immune responses may also be important in continuing amelioration efforts.

Several control efforts have been suggested to mitigate amphibian decline due to chytridiomycosis, such as inoculating ponds with sodium chloride or the use of anti-fungal drugs (Stockwell et al., 2012). Also proposed is probiotic bioaugmentation with protective bacteria known to inhibit *Bd*. Bioaugmentation targets amphibian species that are being released into areas where *Bd* has colonized and where populations are experiencing decline due to chytridiomycosis. All of these approaches though, have seen limitations and lack broad applicability. Therefore, we recommend *in situ* microbiome engineering tools as a novel direction for continued amelioration efforts. Specifically, addition of known antifungal genes and the conferring of protective mechanisms to specific members of a resident bacterial population with plasmid or phage-based genetic modifications may be possible. This is an area our group is currently exploring with great interest for use in the amphibian-disease system and beyond. Recent interest in the literature (Sheth et al., 2016) seems to address this potential use of the bacterial microbiome.

The results and insights provided here will serve as a critical foundation for future studies interested in bacterial responses to *Bd* and continued work on bioaugmentation efforts with *S. marcescens*. Specifically, those genes shown upregulated in both strains of *S. marcescens* would be of interest for further work including the construction of bacterial knockouts as well as complementary studies in *Bd*. Lastly, this data will aid in future and ongoing work addressing manipulation of the amphibian microbiome as well as other disease systems through microbiome engineering efforts.

## Author contributions

JM was involved in the following: anti-Bd assays, RNA sequencing, RT-qPCR, data analysis, and writing the manuscript. EB was involved in the following: anti-Bd assays and related data analysis. JA was involved in the following: isolation and initial characterization of the bacterial isolates and writing the manuscript. OG was involved in the following: RNA sequencing laboratory work. SW was involved in the following: isolation and initial characterization of the bacterial isolates and writing the manuscript. AP was involved in the following: isolation and initial characterization of the bacterial isolates and writing the manuscript. JK was involved in the following: project oversight of all aspects of the study. All authors partook in commenting/editing of the manuscript and have approved the given manuscript for submission. Additionally, all authors agree to be accountable for all aspects of the presented work.

## Funding

This research was done with the support of the United States Fish and Wildlife Service (USFWS Number: 46-6003541) and by the Research Center for Cellular and Molecular Biology, University of Costa Rica (CIBCM Project Number: 801-B2-029). Sequencing data reported in this publication was supported by an Institutional Development Award (IDeA) from the National Institute of General Medicine Sciences of the National Institutes of Health under grant number P20GM103443. Its contents are solely the responsibility of the authors and do not necessarily represent official views of NIGMS or NIH. Support for this research was also supplemented by the John W. Carlson Research grant through the University of South Dakota College of Arts and Sciences.

### Conflict of interest statement

The authors declare that the research was conducted in the absence of any commercial or financial relationships that could be construed as a potential conflict of interest.

## Supplementary material

Additional Supplementary Material for this article can be found online at: http://journal.frontiersin.org/article/10.3389/fmicb.2017.00290/full#supplementary-material

All transcriptome sequencing files are accessible through the NCBI database with accession number GSE84057 and ID 200084057. Included are raw data files, processed data files, and metadata files giving the experimental parameters and conditions. All 16S rDNA sequencing and metadata files are available through the NCBI database with accession numbers KX928035-KX928072. All assembled sequences were checked for chimeras using DECIPHER (Wright et al., 2012). All R scripts used for the generation of graphs and statistical analyses are available through GitHub, (user: kvasir7, repository: dissertation_research).
