# Supplementary material for: Characterization of Batrachochytrium dendrobatidis Inhibiting Bacteria from Amphibian Populations in Costa Rica
Source: Front Microbiol. 2017 Feb 28;8:290. doi: 10.3389/fmicb.2017.00290 (PMC5329008; doi:10.3389/fmicb.2017.00290)
Supplement: Supplementary file 1 [file Table1.DOCX]

|  |  |
| --- | --- |
| Gene | Sequence  **Supplementary Table 1.** Primer sequences utilized in qPCR verification. |
| *chiA*-forward | 5'-GGTGAACGGCTACCAGAACA-3' |
| *chiA*-reverse | 5'-GGCGTCGTAGGTATACTGCC-3' |
| *dnaE*-forward | 5'-GTGGTCAAATGGAAGCCGAC-3' |
| *dnaE*-reverse | 5'-TTGCCGATTTTGCTACCGCT-3' |
| *fadE*-forward | 5'-ATAGGTGATCAGCGAAGCGG-3' |
| *fadE*-reverse | 5'-TCGATTACATCATCGGCGGG-3' |
| *narG*-forward | 5'-ATCAGTTCGCACTCGCCTTT-3' |
| *narG*-reverse | 5'-AAAGCCTTCTCCGAGGTGTG-3' |
| *pigM*-forward | 5'-ACAGGTGGTGCAGGTGAAAA-3' |
| *pigM*-reverse | 5'-CTCGATTTCTCGGATCGCCA-3' |
| *rplU*-forward | 5'-GCTTGGAAAAGCTGGACATC-3' |
| *rplU*-reverse | 5'-TACGGTGGTGTTTACGACGA-3' |
|  |  |
|  |  |
|  |  |
|  |  |
|  |  |
|  |  |
|  |  |
|  |  |
