# Supplementary material for: Characterization of Batrachochytrium dendrobatidis Inhibiting Bacteria from Amphibian Populations in Costa Rica
Source: Front Microbiol. 2017 Feb 28;8:290. doi: 10.3389/fmicb.2017.00290 (PMC5329008; doi:10.3389/fmicb.2017.00290)
Supplement: Supplementary file 2 [file Table2.DOCX]

**Supplementary Table 2:** Comparison of the t values and p values between the mean absorbance measured by a spectrophotometer and the control determined by a GLM analysis of the 90 bacterial isolates examined in the cell-free supernatant challenge assay. (*) represent p values < 0.05. A double asterisk (**) represents amphibians that were sampled but not from relict/recovering populations.

Host Species Isolate t-value p-value

Agalychnis annae B01 -4.85 1.96E-06*

Agalychnis annae B02 -3.734 0.000224*

Agalychnis annae B03 0.821 0.412513

Agalychnis annae B04 0.231 0.817359

Agalychnis annae B05 0.054 0.957161

Agalychnis annae B06 -0.151 0.880145

Agalychnis annae B07 0.433 0.665258

Agalychnis annae B08 1.937 0.053691

Agalychnis annae B09 1.137 0.256359

Agalychnis annae B10 0.239 0.811011

Agalychnis annae B11 0.807 0.420319

Agalychnis annae B12 0.152 0.879285

Agalychnis annae B13 0.586 0.55837

Agalychnis annae B14 1.732 0.084262

Agalychnis annae B15 1.879 0.061129

Agalychnis annae B16 1.893 0.059284

Agalychnis annae B17 -1.84 0.06671

Agalychnis lemur B18 -1.39 0.165577

Agalychnis lemur B19 5.097 6.03E-07*

Agalychnis lemur B20 4.933 1.32E-06*

Agalychnis lemur B21 -0.361 0.718318

Agalychnis lemur B22 1.099 0.27265

Agalychnis lemur B23 0.248 0.804675

Agalychnis lemur B24 0.392 0.69522

Agalychnis lemur B25 0.056 0.954989

Agalychnis lemur B26 0.872 0.383647

Agalychnis lemur B27 0.692 0.489251

Agalychnis lemur B28 -0.094 0.925486

Craugastor bransfordii** B29 0.215 0.830091

Craugastor bransfordii** B30 -0.181 0.856543

Craugastor ranoides B31 -1.704 0.089448

Craugastor bransfordii** B32 -2.151 0.032234*

Craugastor bransfordii** B33 -2.596 0.009881*

Craugastor bransfordii** B34 0.834 0.404794

Craugastor bransfordii** B35 0.346 0.729762

Craugastor bransfordii** B36 -0.844 0.39929

Craugastor bransfordii** B37 -1.63 0.104123

Craugastor bransfordii** B38 -0.271 0.786585

Craugastor bransfordii** B39 -1.562 0.119367

Craugastor bransfordii** B40 0.46 0.645574

Craugastor bransfordii** B41 0.739 0.460637

Craugastor taurus B42 -0.809 0.419378

Craugastor bransfordii** B43 0.056 0.954989

Duellmanohyla rufioculis B44 -0.454 0.650276

Duellmanohyla rufioculis B45 -1.704 0.089448

Duellmanohyla rufioculis B46 0.346 0.729762

Espadarana

prosoblepon** B47 -0.271 0.786585

Hyalinobatrachium

colymbiphyllum** B48 -1.562 0.119367

Incilius holdridgei B49 0.46 0.645574

Incilius holdridgei B50 -1.057 0.291376

Lithobates vibicarius B51 -0.809 0.419378

Lithobates vibicarius B52 -2.836 0.004867*

Lithobates vibicarius B53 0.056 0.954989

Lithobates vibicarius B54 -5.38 1.48E-07*

Oophaga pumilio** B55 0.952 0.342055

Oophaga pumilio** B56 0.253 0.800458

Oophaga pumilio** B57 0.048 0.961507

Oophaga pumilio** B58 0.687 0.492679

Oophaga pumilio** B59 0.406 0.685177

Oophaga pumilio** B60 -0.345 0.730582

Oophaga pumilio** B61 -0.811 0.417812

Oophaga pumilio** B62 0.253 0.800458

Ptychohyla legreri B63 -0.083 0.934154

Ptychohyla legreri B64 0.021 0.983249

Oophaga pumilio** B65 -0.066 0.947172

Oophaga pumilio** B66 0.889 0.374795

Oophaga pumilio** B67 0.482 0.630004

Oophaga pumilio** B68 1.566 0.118469

Agalychnis annae B69 0.758 0.449134

Agalychnis annae B70 0.55 0.582426

Agalychnis lemur B71 1.162 0.246252

Agalychnis lemur B72 -0.336 0.73674

Agalychnis lemur B73 -0.781 0.435229

Agalychnis lemur B74 -0.061 0.951514

Craugastor bransfordii** B75 -1.057 0.291376

Craugastor bransfordii** B76 -0.023 0.981944

Craugastor bransfordii** B77 0.572 0.567564

Craugastor bransfordii** B78 -2.836 0.004867*

Duellmanohyla rufioculis B79 -2.151 0.032234*

Duellmanohyla rufioculis B80 -2.596 0.009881*

Duellmanohyla rufioculis B81 0.834 0.404794

Duellmanohyla rufioculis B82 -0.844 0.39929

Duellmanohyla rufioculis B83 -1.63 0.104123

Lithobates vibicarius B84 0.739 0.460637

Lithobates vibicarius B85 -0.023 0.981944

Lithobates vibicarius B86 0.572 0.567564

Lithobates vibicarius B87 -0.454 0.650276

Oophaga pumilio** B88 -2.637 0.008788*

Oophaga pumilio** B89 0.226 0.821598

Oophaga pumilio** B90 1.489 0.137461
