# Supplementary material for: Characterization of Batrachochytrium dendrobatidis Inhibiting Bacteria from Amphibian Populations in Costa Rica
Source: Front Microbiol. 2017 Feb 28;8:290. doi: 10.3389/fmicb.2017.00290 (PMC5329008; doi:10.3389/fmicb.2017.00290)
Supplement: Supplementary file 3 [file Table3.DOCX]

**Supplementary Table 3:** Comparison of the t values and p values between the mean zone of inhibition (ZOI) measured in cm and the control, where ZOI = 0, determined by a GLM analysis of the 86 bacterial isolates examined in the agar-based assay. Asterisks represent where p < 0.05. A double asterisk (**) represents amphibians that were sampled but not from relict/recovering populations.

Host Species Isolate Mean ZOI (cm^2^) t-value p-value

*Agalychnis annae*  Z01 4.3 4.248 3.16E-05*

*Agalychnis annae*  Z02 3.4 3.311 0.001082*

*Agalychnis annae*  Z03 3.3 3.094 0.002228*

*Agalychnis annae*  Z04 4.3 4.072 6.46E-05*

*Agalychnis annae*  Z05 1.5 1.469 0.143151

*Agalychnis annae*  Z06 4.5 4.405 1.64E-05*

*Agalychnis annae*  Z07 2 1.918 0.056366

*Agalychnis annae*  Z08 2.4 2.236 0.026362*

*Agalychnis annae* Z09 2.2 2.119 0.035169*

*Agalychnis annae* Z10 4.4 4.292 2.63E-05*

*Agalychnis annae*  Z11 4.5 4.43 1.48E-05*

*Agalychnis annae*  Z12 4.5 4.03 7.63E-05*

*Agalychnis annae*  Z13 1.8 1.742 0.082964

*Agalychnis annae*  Z14 1.7 1.629 0.104783

*Agalychnis annae*  Z15 2 2.002 0.046542*

*Agalychnis annae*  Z16 4.6 4.511 1.04E-05*

*Agalychnis annae*  Z17 0.8 0.757 0.449952

*Agalychnis lemur*  Z18 5.6 5.467 1.21E-07*

*Agalychnis lemur* Z19 4 3.905 0.000125*

*Agalychnis lemur* Z20 4 3.902 0.000126*

*Agalychnis lemur*  Z21 4.1 4.204 3.79E-05*

*Agalychnis lemur* Z22 3.4 3.249 0.001336*

*Agalychnis lemur*  Z23 4.2 4.094 5.93E-05*

*Agalychnis lemur*  Z24 1.5 1.457 0.146518

*Agalychnis lemur*  Z25 3.7 3.509 0.000544*

*Agalychnis lemur*  Z26 1.6 1.56 0.120168

*Agalychnis lemur*  Z27 2.6 2.445 0.015272*

*Agalychnis lemur*  Z28 1.1 0.945 0.34572

*Craugastor bransfordii*** Z29 2.1 2.02 0.044543*

*Craugastor bransfordii*** Z30 1.7 1.58 0.115485

*Craugastor ranoides* Z31 0.9 0.881 0.379492

*Craugastor bransfordii*** Z32 1.5 1.431 0.153735

*Craugastor bransfordii*** Z33 2.9 2.838 0.004957*

*Craugastor bransfordii*** Z34 3.2 2.88 0.004369*

*Craugastor bransfordii*** Z35 1.2 1.187 0.236414

*Craugastor bransfordii*** Z36 1.2 1.185 0.237381

*Craugastor bransfordii*** Z37 3.7 3.603 0.000387*

*Craugastor bransfordii*** Z38 1 1.006 0.31566

*Craugastor bransfordii*** Z39 0.8 0.798 0.425723

*Craugastor bransfordii*** Z40 1.5 1.396 0.163953

*Craugastor bransfordii*** Z41 0.3 0.313 0.754196

*Craugastor taurus* Z42 1.9 1.843 0.066654

*Craugastor bransfordii*** Z43 1.5 1.459 0.145843

*Duellmanohyla rufioculis* Z44 1.9 1.852 0.065356

*Duellmanohyla rufioculis* Z45 1.7 1.631 0.104336

*Duellmanohyla rufioculis* Z46 1.9 1.889 0.060225

*Espadarana*

*prosoblepon*** Z47 2.2 2.173 0.030812*

*Hyalinobatrachium*

*colymbiphyllum*** Z48 1.7 1.673 0.095754

*Incilius holdridgei* Z49 0.5 0.473 0.636389

*Incilius holdridgei* Z50 3.4 3.243 0.001365*

*Lithobates vibicarius* Z51 1.8 1.766 0.078749

*Lithobates vibicarius* Z52 3.1 3.088 0.002269*

*Lithobates vibicarius* Z53 2 1.938 0.05391

*Lithobates vibicarius* Z54 1.2 1.18 0.239323

*Oophaga pumilio*** Z55 2.3 2.271 0.024076*

*Oophaga pumilio*** Z56 1.9 1.818 0.070467

*Oophaga pumilio*** Z57 2.4 2.15 0.032661*

*Oophaga pumilio*** Z58 0.4 0.356 0.722427

*Oophaga pumilio*** Z59 2.5 2.497 0.013245*

*Oophaga pumilio*** Z60 1.1 1.058 0.291354

*Oophaga pumilio*** Z61 4.2 4.084 6.16E-05*

*Oophaga pumilio*** Z62 1.6 1.51 0.132331

*Ptychohyla legreri* Z63 1.7 1.619 0.10688

*Ptychohyla legreri* Z64 0 0 1

*Oophaga pumilio*** Z65 2.4 2.357 0.019276*

*Oophaga pumilio*** Z66 3.7 3.584 0.000416*

*Oophaga pumilio*** Z67 5.1 5.048 9.24E-07*

*Oophaga pumilio*** Z68 1.5 1.499 0.135361

*Agalychnis annae* Z91 1.7 1.646 0.101194

*Agalychnis annae* Z92 5.3 5.183 4.87E-07*

*Agalychnis annae*  Z93 4.7 4.616 6.58E-06*

*Agalychnis annae*  Z94 4.5 4.256 3.06E-05*

*Agalychnis annae* Z95 1.7 1.583 0.114762

*Agalychnis annae*  Z96 1.9 1.818 0.070455

*Agalychnis annae* Z97 1.4 1.336 0.182803

*Agalychnis lemur* Z98 0.7 0.638 0.524293

*Agalychnis lemur*  Z99 0.6 0.623 0.533902

*Agalychnis lemur*  Z100 1 0.457 0.648269

*Agalychnis lemur*  Z101 1.3 1.268 0.20607

*Craugastor ranoides* Z102 0.8 0.779 0.436809

*Oophaga pumilio*** Z103 1.8 1.798 0.073533

*Oophaga pumilio*** Z104 0.6 0.562 0.57488

*Ptychohyla legleri* Z105 1.2 1.163 0.246208

*Ptychohyla legleri* Z106 1.3 1.31 0.191597

*Ptychohyla legleri* Z107 2.5 2.45 0.015036*
