# Supplementary material for: Characterization of Batrachochytrium dendrobatidis Inhibiting Bacteria from Amphibian Populations in Costa Rica
Source: Front Microbiol. 2017 Feb 28;8:290. doi: 10.3389/fmicb.2017.00290 (PMC5329008; doi:10.3389/fmicb.2017.00290)
Supplement: Supplementary file 4 [file Table4.DOCX]

**Supplementary Table 4:** Comparison between bacterial isolates that exhibited anti-*Bd* properties where p < 0.05 in cell-free supernatant challenge assay and agar-based challenge assay represented by asterisks (*). The host of each bacterial isolate is included. For bacterial isolates that were sequenced, bacterial species is included. A double asterisk (**) represents amphibians that were sampled but not from relict/recovering populations.

| Amphibian Host | Bacterial Species | Cell-Free | Inhibit | Enhance | Agar | Inhibit |
| --- | --- | --- | --- | --- | --- | --- |
| *Agalychnis annae* |  | B01 | * |  | Z01 | * |
| *Agalychnis annae* |  | B02 | * |  | Z02 | * |
| *Agalychnis annae* |  | B03 |  |  | Z03 | * |
| *Agalychnis annae* | *Serratia marcescens* | B04 |  |  | Z04 | * |
| *Agalychnis annae* |  | B05 |  |  | Z05 |  |
| *Agalychnis annae* | *Serratia marcescens* | B06 |  |  | Z06 | * |
| *Agalychnis annae* | *Serratia sp.* | B07 |  |  | Z07 |  |
| *Agalychnis annae* | *Pseudomonas fulva* | B08 |  |  | Z08 | * |
| *Agalychnis annae* |  | B09 |  |  | Z09 | * |
| *Agalychnis annae* |  | B10 |  |  | Z10 | * |
| *Agalychnis annae* | *Serratia marcescens* | B11 |  |  | Z11 | * |
| *Agalychnis annae* | *Serratia marcescens* | B12 |  |  | Z12 | * |
| *Agalychnis annae* |  | B13 |  |  | Z13 |  |
| *Agalychnis annae* |  | B14 |  |  | Z14 |  |
| *Agalychnis annae* | *Enterobacteriaceae bacterium* | B15 |  |  | Z15 | * |
| *Agalychnis annae* |  | B16 |  |  | Z16 | * |
| *Agalychnis annae* | *Serratia sp.* | B17 |  |  | Z17 |  |
| *Agalychnis lemur* | *Serratia marcescens* | B18 |  |  | Z18 | * |
| *Agalychnis lemur* | *Serratia marcescens* | B19 |  | * | Z19 | * |
| *Agalychnis lemur* | *Serratia marcescens* | B20 |  | * | Z20 | * |
| *Agalychnis lemur* | *Serratia sp.* | B21 |  |  | Z21 | * |
| *Agalychnis lemur* | *Serratia marcescens* | B22 |  |  | Z22 | * |
| *Agalychnis lemur* | *Serratia sp.* | B23 |  |  | Z23 | * |
| *Agalychnis lemur* |  | B24 |  |  | Z24 |  |
| *Agalychnis lemur* | *Serratia sp.* | B25 |  |  | Z25 | * |
| *Agalychnis lemur* | *Microbacterium keratanolyticum* | B26 |  |  | Z26 |  |
| *Agalychnis lemur* |  | B27 |  |  | Z27 | * |
| *Agalychnis lemur* |  | B28 |  |  | Z28 |  |
| *Craugastor bransfordii*** |  | B29 |  |  | Z29 | * |
| *Craugastor bransfordii*** |  | B30 |  |  | Z30 |  |
| *Craugastor ranoides* |  | B31 |  |  | Z31 |  |
| *Craugastor bransfordii*** |  | B32 | * |  | Z32 |  |
| *Craugastor bransfordii*** |  | B33 | * |  | Z33 | * |
| *Craugastor bransfordii*** |  | B34 |  |  | Z34 | * |
| *Craugastor bransfordii*** |  | B35 |  |  | Z35 |  |
| *Craugastor bransfordii*** |  | B36 |  |  | Z36 |  |
| *Craugastor bransfordii*** | *Chyrseobacterium sp.* | B37 |  |  | Z37 | * |
| *Craugastor bransfordii*** |  | B38 |  |  | Z38 |  |
| *Craugastor bransfordii*** |  | B39 |  |  | Z39 |  |
| *Craugastor bransfordii*** |  | B40 |  |  | Z40 |  |
| *Craugastor bransfordii*** |  | B41 |  |  | Z41 |  |
| *Craugastor taurus* | *Alcaligenes faecalis* | B42 |  |  | Z42 |  |
| *Craugastor bransfordii*** |  | B43 |  |  | Z43 |  |
| *Duellmanohyla rufioculis* |  | B44 |  |  | Z44 |  |
| *Duellmanohyla rufioculis* |  | B45 |  |  | Z45 |  |
| *Duellmanohyla rufioculis* |  | B46 |  |  | Z46 |  |
| *Espadarana prosoblepon*** |  | B47 |  |  | Z47 | * |
| *Hyalinobatrachium colymbiphyllum*** |  | B48 |  |  | Z48 |  |
| *Incilius holdridgei* | *Sphingobacterium sp.* | B49 |  |  | Z49 |  |
| *Incilius holdridgei* |  | B50 |  |  | Z50 | * |
| *Lithobates vibicarius* |  | B51 |  |  | Z51 |  |
| *Lithobates vibicarius* |  | B52 | * |  | Z52 | * |
| *Lithobates vibicarius* | *Bacillus sp.* | B53 |  |  | Z53 |  |
| *Lithobates vibicarius* | *Stenotrophomonas* | B54 | * |  | Z54 |  |
| *Oophaga pumilio*** |  | B55 |  |  | Z55 | * |
| *Oophaga pumilio*** |  | B56 |  |  | Z56 |  |
| *Oophaga pumilio*** | *Chryseobacterium vietnamense* | B57 |  |  | Z57 | * |
| *Oophaga pumilio*** | *Lysinibacillus fusiformis* | B58 |  |  | Z58 |  |
| *Oophaga pumilio*** |  | B59 |  |  | Z59 | * |
| *Oophaga pumilio*** | *Chryseobacterium vietnamense* | B60 |  |  | Z60 |  |
| *Oophaga pumilio*** |  | B61 |  |  | Z61 | * |
| *Oophaga pumilio*** | *Stenotrophomonas maltophilia* | B62 |  |  | Z62 |  |
| *Ptychohyla legreri* |  | B63 |  |  | Z63 |  |
| *Ptychohyla legreri* |  | B64 |  |  | Z64 |  |
| *Oophaga pumilio*** |  | B65 |  |  | Z65 | * |
| *Oophaga pumilio*** |  | B66 |  |  | Z66 | * |
| *Oophaga pumilio*** | *Serratia marcescens* | B67 |  |  | Z67 | * |
| *Oophaga pumilio*** |  | B68 |  |  | Z68 |  |
| *Agalychnis annae* |  | B69 |  |  |  |  |
| *Agalychnis annae* | *Chyseobacterium sp.* | B70 |  |  |  |  |
| *Agalychnis lemur* |  | B71 |  |  |  |  |
| *Agalychnis lemur* |  | B72 |  |  |  |  |
| *Agalychnis lemur* |  | B73 |  |  |  |  |
| *Agalychnis lemur* |  | B74 |  |  |  |  |
| *Craugastor bransfordii*** |  | B75 |  |  |  |  |
| *Craugastor bransfordii*** |  | B76 |  |  |  |  |
| *Craugastor bransfordii*** |  | B77 |  |  |  |  |
| *Craugastor bransfordii*** |  | B78 | * |  |  |  |
| *Duellmanohyla rufioculis* |  | B79 | * |  |  |  |
| *Duellmanohyla rufioculis* |  | B80 | * |  |  |  |
| *Duellmanohyla rufioculis* |  | B81 |  |  |  |  |
| *Duellmanohyla rufioculis* |  | B82 |  |  |  |  |
| *Duellmanohyla rufioculis* |  | B83 |  |  |  |  |
| *Lithobates vibicarius* |  | B84 |  |  |  |  |
| *Lithobates vibicarius* |  | B85 |  |  |  |  |
| *Lithobates vibicarius* |  | B86 |  |  |  |  |
| *Lithobates vibicarius* |  | B87 |  |  |  |  |
| *Oophaga pumilio*** |  | B88 | * |  |  |  |
| *Oophaga pumilio*** |  | B89 |  |  |  |  |
| *Oophaga pumilio*** |  | B90 |  |  |  |  |
| *Agalychnis annae* | *Chyrseobacterium* |  |  |  | Z91 |  |
| *Agalychnis annae* | *Serratia sp.* |  |  |  | Z92 | * |
| *Agalychnis annae* |  |  |  |  | Z93 | * |
| *Agalychnis annae* |  |  |  |  | Z94 | * |
| *Agalychnis annae* | *Staphylococcus equorum* |  |  |  | Z95 |  |
| *Agalychnis annae* | *Bacillus mycoides* |  |  |  | Z96 |  |
| *Agalychnis annae* | *Stenotrophomonas* |  |  |  | Z97 |  |
| *Agalychnis lemur* | *Bacillus sp.* |  |  |  | Z98 |  |
| *Agalychnis lemur* |  |  |  |  | Z99 |  |
| *Agalychnis lemur* | *Stenotrophomonas* |  |  |  | Z100 |  |
| *Agalychnis lemur* | *Staphyloccoccus xylosus* |  |  |  | Z101 |  |
| *Craugastor ranoides* |  |  |  |  | Z102 |  |
| *Oophaga pumilio*** |  |  |  |  | Z103 |  |
| *Oophaga pumilio*** |  |  |  |  | Z104 |  |
| *Ptychohyla legleri* |  |  |  |  | Z105 |  |
| *Ptychohyla legleri* |  |  |  |  | Z106 |  |
| *Ptychohyla legleri* |  |  |  |  | Z107 | * |
