# Supplementary material for: Characterization of Batrachochytrium dendrobatidis Inhibiting Bacteria from Amphibian Populations in Costa Rica
Source: Front Microbiol. 2017 Feb 28;8:290. doi: 10.3389/fmicb.2017.00290 (PMC5329008; doi:10.3389/fmicb.2017.00290)
Supplement: Supplementary file 5 [file Table5.DOCX]

| Sequenced Isolates | Total Isolates |
| --- | --- |
| *Alcaligenes faecalis* | 1 |
| *Bacillus mycoides* | 1 |
| *Bacillus* sp*.* | 1 |
| *Chryseobacterium vietnamense* | 2 |
| *Chyrseobacterium* sp*.* | 3 |
| *Enterobacteriaceae bacterium* | 1 |
| *Lysinibacillus fusiformis* | 1 |
| *Microbacterium keratanolyticum* | 1 |
| *Pseudomonas fulva* | 1 |
| *Serratia marcesens* | 9 |
| *Serratia* sp*.* | 6 |
| *Sphingobacterium* sp*.* | 1 |
| *Staphyloccoccus xylosus* | 1 |
| *Staphylococcus equorum* | 1 |
| *Stenotrophomonas maltophilia* | 1 |
| *Stenotrophomonas* sp*.* | 3 |

**Supplementary Table 5:** Anti-*Bd* bacteria identified through 16S rDNA sequencing.
