# Supplementary material for: Characterization of Batrachochytrium dendrobatidis Inhibiting Bacteria from Amphibian Populations in Costa Rica
Source: Front Microbiol. 2017 Feb 28;8:290. doi: 10.3389/fmicb.2017.00290 (PMC5329008; doi:10.3389/fmicb.2017.00290)
Supplement: Supplementary file 6 [file Table6.DOCX]

**Supplementary Table 6.** Genes significantly up and down-regulated (q-value<0.05) from *S. marcescens* strain one with growth in the presence of live *Bd*. Not all genes and associated products/function were identified when compared to the WW4 reference genome and are indicated by a hyphen (-).

|  |  |  |
| --- | --- | --- |
| Gene Name | Product | Fold Change *Bd* vs Control |
| paaA | phenylacetate-CoA oxygenase subunit PaaA | 0.071429 |
| fadE | acyl coenzyme A dehydrogenase | 0.08377 |
| kgtP | alpha-ketoglutarate transporter | 0.129032 |
| hisQ | histidine/lysine/arginine/ornithine transporter permease subunit | 0.142857 |
| dppB | dipeptide/heme transporter | 0.15625 |
| - | hypothetical protein | 0.16129 |
| dppD | dipeptide/heme transporter | 0.166667 |
| fadB | multifunctional fatty acid oxidation complex subunit alpha | 0.175 |
| - | hypothetical protein | 0.202937 |
| - | hypothetical protein | 0.218354 |
| - | lactate permease family transporter | 0.220588 |
| astC | succinylornithine transaminase, PLP-dependent | 0.22449 |
| - | Rieske (2Fe-2S) domain-containing protein | 0.230769 |
| - | cupin | 0.230769 |
| - | - | 0.235405 |
| argT | lysine/arginine/ornithine transporter subunit | 0.238532 |
| acs | acetyl-CoA synthetase | 0.238938 |
| putP | proline:sodium symporter | 0.25 |
| - | dipeptide ABC transporter periplasmic dipeptide-binding protein | 0.254513 |
| fadD | acyl-CoA synthetase | 0.256098 |
| rsd | stationary phase protein | 0.261603 |
| - | antisense: SMWW4_v1r110 | 0.262821 |
| - | antisense: SMWW4_v1r020 | 0.262857 |
| - | antisense: SMWW4_v1r150 | 0.262857 |
| - | antisense: SMWW4_v1r080 | 0.262857 |
| - | antisense: SMWW4_v1r050 | 0.264368 |
| - | antisense: SMWW4_v1r210 | 0.267045 |
| - | gluconate 2-dehydrogenase | 0.268293 |
| - | universal stress protein A | 0.272727 |
| - | antisense: SMWW4_v1r180 | 0.273292 |
| fadJ | multifunctional fatty acid oxidation complex subunit alpha | 0.285714 |
| - | citrate carrier protein | 0.291667 |
| - | Rieske (2Fe-2S) domain-containing protein | 0.295833 |
| fadI | 3-ketoacyl-CoA thiolase | 0.307692 |
| astD | succinylglutamic semialdehyde dehydrogenase | 0.333333 |
| gltI | glutamate, aspartate binding protein, periplasmic | 0.339965 |
| amtB | ammonium transporter | 0.344828 |
| putA | proline dehydrogenase / delta 1-pyrroline-5-carboxylate dehydrogenase | 0.350962 |
| - | hypothetical protein | 0.352941 |
| glpD | FAD/NAD(P)-binding sn-glycerol-3-phosphate dehydrogenase | 0.355183 |
| - | hypothetical protein | 0.356902 |
| - | IclR family transcriptional regulator | 0.361702 |
| betI | DNA-binding transcriptional repressor | 0.363636 |
| - | amino acid/peptide transporter | 0.37013 |
| yohC | inner membrane protein, Yip1 family | 0.377778 |
| - | hypothetical protein | 0.383553 |
| - | urocanate hydratase | 0.38671 |
| - | hypothetical protein | 0.392405 |
| sdhC | succinate dehydrogenase, cytochrome b556 large membrane subunit | 0.414333 |
| - | hypothetical protein | 0.414634 |
| dctA | C4-dicarboxylic acid, orotate and citrate transporter | 0.421659 |
| acnA | aconitate hydratase 1 | 0.459259 |
| oppA1 | oligopeptide ABC transporter substrate-binding protein OppA1 | 0.466667 |
| ybaY | outer membrane lipoprotein | 0.485493 |
| yegQ | putative peptidase | 0.496241 |
| - | Histidine ammonia-lyase | 0.496403 |
| - | hypothetical protein, UPF0267 family | 0.49734 |
| - | aldehyde oxidase and xanthine dehydrogenase | 0.505747 |
| - | - | 0.810492 |
| - | antisense: aspA | 2.083333 |
| mug | G/U mismatch-specific DNA glycosylase | 2.09375 |
| - | antisense: rplD | 2.114407 |
| - | hypothetical protein, UPF0758 family | 2.114754 |
| cdgR | putative cyclic di-GMP regulator, inactive EAL family phosphodiesterase | 2.116279 |
| - | antisense: rpsS | 2.146199 |
| malF | maltose transporter subunit | 2.181818 |
| - | antisense: frdA | 2.203488 |
| - | antisense: SMWW4_v1c45710 | 2.325137 |
| - | cold-shock DNA-binding domain-containing protein | 2.360549 |
| - | - | 2.38446 |
| - | beta-N-acetylhexosaminidase | 2.384615 |
| - | L-serine dehydratase 1 | 2.415254 |
| gpmM | phosphoglycero mutase III, cofactor-independent | 2.441176 |
| mutT | nucleoside triphosphate pyrophosphohydrolase, marked preference for dGTP | 2.449275 |
| - | MarR family transcriptional regulator | 2.473684 |
| ybjM | inner membrane protein | 2.504274 |
| - | antisense: SMWW4_v1c16850 | 2.514151 |
| - | protein tyrosine/serine phosphatase | 2.5625 |
| ccmE | cytochrome c-type biogenesis protein CcmE | 2.571429 |
| - | antisense: ptsI | 2.619335 |
| focA | formate channel | 2.64467 |
| - | - | 2.729592 |
| - | antisense: aspA | 2.78 |
| - | - | 2.819672 |
| - | antisense: secY | 2.840278 |
| - | ornithine decarboxylase | 2.844037 |
| fliI | flagellum-specific ATP synthase | 2.875 |
| citX | apo-citrate lyase phosphoribosyl-dephospho-CoA transferase | 2.875 |
| - | methylmalonate-semialdehyde dehydrogenase | 2.9 |
| citE | citrate lyase, citryl-ACP lyase beta subunit | 2.95 |
| ptsG | glucose-specific PTS system IIBC component | 2.96063 |
| - | antisense: fbaA | 2.969925 |
| - | alpha-acetolactate decarboxylase | 3.105263 |
| - | antisense: fusA2 | 3.190476 |
| feoA | ferrous iron transporter, protein A | 3.208333 |
| citT | citrate:succinate antiporter | 3.307692 |
| fruA | PTS system fructose-specific transporter subunits IIBC | 3.444444 |
| - | hypothetical protein | 3.5 |
| maa | maltose O-acetyltransferase | 3.573529 |
| - | putative transcriptional regulator | 3.580645 |
| adhE | bifunctional acetaldehyde-CoA/alcohol dehydrogenase | 3.657658 |
| - | antisense: treC | 3.943522 |
| yobD | inner membrane protein, UPF0266 family | 3.947368 |
| - | - | 4.091429 |
| pykF | pyruvate kinase I | 4.096774 |
| - | antisense: malE | 4.134831 |
| treB | trehalose(maltose)-specific PTS system IIBC component | 4.216157 |
| fruK | fructose-1-phosphate kinase | 4.6 |
| dpiA | DNA-binding response regulator in two-component regulatory system with citA | 5.166667 |
| - | antisense: adhE | 6.579545 |
| - | HxlR family transcriptional regulator | 7.333333 |
