# Supplementary material for: Characterization of Batrachochytrium dendrobatidis Inhibiting Bacteria from Amphibian Populations in Costa Rica
Source: Front Microbiol. 2017 Feb 28;8:290. doi: 10.3389/fmicb.2017.00290 (PMC5329008; doi:10.3389/fmicb.2017.00290)
Supplement: Supplementary file 7 [file Table7.DOCX]

**Supplementary Table 7.** Genes significantly up and down-regulated (q-value<0.05) from *S. marcescens* strain two with growth in the presence of *Bd*. Not all genes and associated products/function were identified when compared to the WW4 reference genome and are indicated by a hyphen (-).

| Gene Name | Product |  | Fold Change *Bd* vs Control |
| --- | --- | --- | --- |
| - | hypothetical protein |  | 0.04 |
| tadA | tRNA-specific adenosine deaminase |  | 0.086957 |
| - | antisense: rplQ |  | 0.231285 |
| ygaH | putative amino acid transporter YgaH |  | 0.25 |
| - | hypothetical protein |  | 0.272727 |
| - | hypothetical protein, UPF0178 family |  | 0.285714 |
| - | antisense: frr |  | 0.289179 |
| - | antisense: cyoA |  | 0.304745 |
| - | antisense: cmk |  | 0.315978 |
| - | antisense: rho |  | 0.319285 |
| - | antisense: SMWW4_v1c16980 |  | 0.325116 |
| - | antisense: treC |  | 0.330508 |
| - | putative cysteine methyltransferase |  | 0.333333 |
| - | antisense: atpA |  | 0.346715 |
| trxC | thioredoxin 2 |  | 0.36 |
| - | antisense: ahpC |  | 0.360223 |
| - | - |  | 0.369089 |
| - | antisense: lpd |  | 0.374825 |
| osmE | DNA-binding transcriptional activator |  | 0.378378 |
| - | antisense: SMWW4_v1c35100 |  | 0.380435 |
| - | two component LuxR family transcriptional regulator |  | 0.384615 |
| yebN | inner membrane protein |  | 0.391304 |
| - | AraC family transcriptional regulator |  | 0.4 |
| - | antisense: typA |  | 0.408537 |
| ybaN | inner membrane protein, DUF454 family |  | 0.409091 |
| - | antisense: rpoA rplQ |  | 0.418447 |
| - | antisense: rplC rpsJ |  | 0.418729 |
| - | cupin |  | 0.419355 |
| ydgI | putative arginine/ornithine antiporter transporter |  | 0.423529 |
| - | membrane-bound beta-hydroxylase |  | 0.424242 |
| - | LysR family transcriptional regulator |  | 0.428571 |
| purE | N5-carboxyaminoimidazole ribonucleotide mutase |  | 0.428571 |
| artQ | arginine ABC transporter permease ArtQ |  | 0.433333 |
| - | antisense: rplK |  | 0.440967 |
| - | antisense: rpsD |  | 0.441672 |
| sugE | multidrug efflux system protein |  | 0.442857 |
| - | antisense: cyoB |  | 0.451056 |
| bioC | malonyl-CoA methyltransferase, SAM-dependent |  | 0.457143 |
| - | antisense: ybgF pal |  | 0.464052 |
| - | antisense: rpsA |  | 0.464403 |
| - | antisense: rpmF SMWW4_v1c18600 |  | 0.464811 |
| folA | dihydrofolate reductase |  | 0.465986 |
| - | antisense: secY |  | 0.469244 |
| - | lysine exporter protein LysE/YggA |  | 0.470588 |
| - | antisense: ptsI |  | 0.476404 |
| - | antisense: rplA |  | 0.483483 |
| ubiX | 3-octaprenyl-4-hydroxybenzoate carboxy-lyase |  | 0.484848 |
| - | hypothetical protein |  | 0.5 |
| - | hypothetical protein |  | 0.504762 |
| - | antisense: rplJ |  | 0.506494 |
| - | antisense: rplA |  | 0.509579 |
| - | antisense: rpsQ |  | 0.510029 |
| - | antisense: hupA |  | 0.512821 |
| - | antisense: rplM |  | 0.520716 |
| - | antisense: SMWW4_v1c30770 |  | 0.521183 |
| - | antisense: rpsE |  | 0.521368 |
| - | hypothetical protein |  | 0.52381 |
| aaeA | p-hydroxybenzoic acid efflux system subunit AaeA |  | 0.52381 |
| - | antisense: rpoB |  | 0.524501 |
| - | phage holin |  | 0.533333 |
| yajG | putative lipoprotein |  | 0.541284 |
| yfhH | putative DNA-binding transcriptional regulator |  | 0.541667 |
| - | antisense: ptsI |  | 0.556452 |
| - | antisense: atpE atpB |  | 0.584122 |
| - | colicin-like protein |  | 0.588028 |
| ldhA | fermentative D-lactate dehydrogenase, NAD-dependent |  | 1.865546 |
| manX | mannose-specific PTS system IIA component |  | 1.934066 |
| - | hypothetical protein |  | 2.09375 |
| - | GntR family transcriptional regulator |  | 2.109589 |
| yfiD | autonomous glycyl radical cofactor |  | 2.174609 |
| bssS | biofilm regulator |  | 2.213942 |
| torC | trimethylamine N-oxide (TMAO) reductase I, cytochrome c-type subunit |  | 2.233333 |
| ybhI | putative transporter |  | 2.27027 |
| - | - |  | 2.312139 |
| yfbT | putative phosphatase |  | 2.363636 |
| bfd | bacterioferritin-associated ferredoxin |  | 2.393258 |
| - | hypothetical protein, UPF0304 family |  | 2.414414 |
| mepA | murein DD-endopeptidase |  | 2.4375 |
| yhbU | putative peptidase (collagenase-like) |  | 2.475 |
| - | - |  | 2.524675 |
| cydB | cytochrome d terminal oxidase, subunit II |  | 2.585366 |
| - | hypothetical protein |  | 2.602469 |
| - | - |  | 2.719626 |
| feoB | ferrous iron transporter, protein B |  | 3 |
| nirB | NAD(P)H-binding nitrite reductase, large subunit |  | 3.004717 |
| cadB | putative lysine/cadaverine transporter |  | 3.318182 |
| ilvC | NAD(P)-binding ketol-acid reductoisomerase |  | 3.362694 |
| frdB | fumarate reductase, Fe-S subunit |  | 3.468085 |
| menB | dihydroxynaphthoic acid synthetase |  | 3.543478 |
| - | lysine decarboxylase |  | 3.696682 |
| uxuA | mannonate hydrolase |  | 3.833333 |
| frdC | fumarate reductase, membrane anchor subunit |  | 4.321429 |
| menE | o-succinylbenzoate-CoA ligase |  | 4.5 |
| - | nitrite transporter |  | 7.5 |
| cysG | siroheme synthase |  | 9.833333 |
| - | nitrate reductase, alpha subunit |  | 14 |
| - | ornithine decarboxylase |  | 20 |
| - | - |  | 20.48276 |
| - | nitrate reductase, beta subunit |  | 28 |
| yhjX | Inner membrane protein, putative oxalate-formate antiporter |  | 28.66667 |
