# Supplementary material for: Characterization of Batrachochytrium dendrobatidis Inhibiting Bacteria from Amphibian Populations in Costa Rica
Source: Front Microbiol. 2017 Feb 28;8:290. doi: 10.3389/fmicb.2017.00290 (PMC5329008; doi:10.3389/fmicb.2017.00290)
Supplement: Supplementary file 8 [file Table8.DOCX]

**Supplementary Table 8.** Genes significantly up and down-regulated (q-value<0.05) from *S. marcescens* strain one with growth in the presence of heat-killed *Bd*. Not all genes and associated products/function were identified when compared to the WW4 reference genome and are indicated by a hyphen (-).

| Gene Name | Product | Fold Change HKBd vs Control |
| --- | --- | --- |
| amtB | ammonium transporter | 0.275862 |
| glnA | glutamine synthetase | 0.331092 |
| - | hypothetical protein | 0.347826 |
| glpD | FAD/NAD(P)-binding sn-glycerol-3-phosphate dehydrogenase | 0.384909 |
| - | hypothetical protein | 0.416667 |
| pyrC | dihydro-orotase | 0.425926 |
| psd | phosphatidylserine decarboxylase | 0.432432 |
| lysA | PLP-binding diaminopimelate decarboxylase | 0.438202 |
| lolC | lipoprotein-releasing system transmembrane protein | 0.478261 |
| purM | phosphoribosylaminoimidazole synthetase | 0.479554 |
| dacB | D-alanyl-D-alanine carboxypeptidase | 0.483871 |
| purR | hypoxanthine-binding transcriptional repressor | 0.483871 |
| yceJ | putative cytochrome b561 | 0.484848 |
| glnP | glutamine ABC transporter permease | 0.486486 |
| - | hypothetical protein | 0.494118 |
| - | dihydrodipicolinate synthase | 0.5 |
| - | hypothetical protein | 0.5 |
| - | tRNA-hydroxylase | 0.5 |
| rimN | tRNA(ANN) t(6)A37 threonylcarbamoyladenosine modification protein | 0.510204 |
| fmt | 10-formyltetrahydrofolate:L-methionyl-tRNA(fMet) N-formyltransferase | 0.513514 |
| folB | bifunctional dihydroneopterin aldolase/dihydroneopterin triphosphate 2'-epimerase | 0.516129 |
| yrfG | GMP/IMP nucleotidase | 0.518072 |
| purH | bifunctional phosphoribosylaminoimidazolecarboxamide formyltransferase/IMP cyclohydrolase | 0.521739 |
| yegQ | putative peptidase | 0.533835 |
| rfaQ | lipopolysaccharide core biosynthesis protein | 0.535714 |
| - | - | 0.537313 |
| trmA | tRNA m(5)U54 methyltransferase, SAM-dependen | 0.55 |
| can | carbonic anhydrase | 0.55102 |
| - | Ile tRNA | 0.554187 |
| ispF | 2C-methyl-D-erythritol 2,4-cyclodiphosphate synthase | 0.556452 |
| betI | DNA-binding transcriptional repressor | 0.558442 |
| tsgA | putative transporter | 0.559322 |
| putP | proline:sodium symporter | 0.5625 |
| ydiY | putative outer membrane protein, acid-inducible | 0.5625 |
| msbA | lipid A export permease/ATP-binding protein MsbA | 0.564103 |
| - | Ile tRNA | 0.56691 |
| emrD | multidrug efflux system protein | 0.571429 |
| - | thiamine pyrophosphate binding domain-containing protein | 0.571429 |
| yheT | putative hydrolase | 0.571429 |
| tatD | TatD DNase family protein | 0.573333 |
| - | Rrf2 family transcriptional regulator | 0.57377 |
| rnd | ribonuclease D | 0.57377 |
| smrB | putative DNA endonuclease | 0.574468 |
| rcsF | putative outer membrane protein, signal | 0.576923 |
| hemG | protoporphyrin oxidase, flavoprotein | 0.580247 |
| - | hypothetical protein | 0.586957 |
| purN | phosphoribosylglycinamide formyltransferase 1 | 0.596154 |
| gpt | guanine-hypoxanthine phosphoribosyltransferase | 0.6 |
| - | AMP-dependent synthetase and ligase | 0.6 |
| malF | maltose transporter subunit | 0.6 |
| rsmI | 16S rRNA C1402 ribose 2'-O-methyltransferase, SAM-dependent | 0.603774 |
| proY | proline-specific permease | 0.608696 |
| yqhC | transcriptional activator of yqhD | 0.617647 |
| iap | aminopeptidase in alkaline phosphatase isozyme conversion | 0.62069 |
| trkH | potassium transporter | 0.625 |
| thyA | thymidylate synthetase | 0.625 |
| - | Thr tRNA | 0.630072 |
| - | aldehyde dehydrogenase | 0.636364 |
| rsmC | 16S rRNA m(2)G1207 methyltransferase | 0.636364 |
| - | carbon starvation protein CstA | 0.673777 |
| - | antisense: infC rpmI | 1.899756 |
| - | - | 1.978667 |
| - | antisense: hupB | 2.114058 |
| - | DoxX family protein | 2.117557 |
| - | hypothetical protein | 2.127273 |
| ulaC | L-ascorbate-specific enzyme IIA component of PTS | 2.19939 |
| - | antisense: secY | 2.275568 |
| - | - | 2.348457 |
| - | ABC-type branched-chain amino acid transport system, periplasmic component | 2.391304 |
| - | antisense: raiA | 2.420054 |
| - | sugar ABC transporter periplasmic protein | 2.441558 |
| - | antisense: cspC | 2.446456 |
| - | antisense: aspA | 2.479821 |
| - | antisense: ompA | 2.52682 |
| - | - | 2.566964 |
| - | - | 2.697115 |
| - | thioesterase superfamily protein | 3 |
| ygbJ | putative dehydrogenase | 3 |
| citT | citrate:succinate antiporter | 3.153846 |
| - | antisense: secY | 3.201389 |
| - | antisense: aspA | 3.26 |
| - | antisense: yeaG | 3.628571 |
| - | antisense: ychH | 3.671141 |
| citC | [citrate [pro-3S]-lyase] ligase | 3.814815 |
| - | glutathione S-transferase | 4.166667 |
| citE | citrate lyase, citryl-ACP lyase beta subunit | 4.2 |
| - | citrate carrier protein | 4.416667 |
| - | antisense: SMWW4_v1r210 | 4.573864 |
| - | antisense: SMWW4_v1r020 | 4.6 |
| - | antisense: SMWW4_v1r150 | 4.6 |
| - | antisense: SMWW4_v1r080 | 4.6 |
| - | antisense: SMWW4_v1r180 | 4.602484 |
| - | antisense: SMWW4_v1r050 | 4.632184 |
| citF | citrate lyase, citrate-ACP transferase alpha subunit | 4.666667 |
| - | antisense: SMWW4_v1r110 | 4.788462 |
| rpmJ | 50S ribosomal subunit protein L36 | 5.125 |
