# Supplementary material for: Characterization of Batrachochytrium dendrobatidis Inhibiting Bacteria from Amphibian Populations in Costa Rica
Source: Front Microbiol. 2017 Feb 28;8:290. doi: 10.3389/fmicb.2017.00290 (PMC5329008; doi:10.3389/fmicb.2017.00290)
Supplement: Supplementary file 9 [file Table9.DOCX]

**Supplementary Table 9.** Genes significantly up and down-regulated (q-value<0.05) from *S. marcescens* strain two with growth in the presence of heat-killed *Bd*. Not all genes and associated products/function were identified when compared to the WW4 reference genome and are indicated by a hyphen (-).

| Gene Name | Product |  |  | Fold Change HKBd vs Control |
| --- | --- | --- | --- | --- |
| yhdT | inner membrane protein |  |  | 0.121212 |
| - | hypothetical protein |  |  | 0.148352 |
| - | putative cysteine methyltransferase |  |  | 0.166667 |
| artQ | arginine ABC transporter permease ArtQ |  |  | 0.2 |
| rnfB | electron transport complex protein RnfB |  |  | 0.235294 |
| - | ferric iron ABC transporter, permease protein |  |  | 0.24 |
| lysP | lysine transporter |  |  | 0.258772 |
| ydgI | putative arginine/ornithine antiporter transporter |  |  | 0.258824 |
| bioC | malonyl-CoA methyltransferase, SAM-dependent |  |  | 0.285714 |
| yqhA | putative inner membrane protein, UPF0114 family |  |  | 0.291667 |
| - | - |  |  | 0.297983 |
| suhB | inositol monophosphatase |  |  | 0.298009 |
| metN | D-methionine transport system ATP-binding protein |  |  | 0.3 |
| - | hypothetical protein |  |  | 0.302013 |
| - | Ser tRNA |  |  | 0.302752 |
| - | xanthine/uracil/vitamin C permease |  |  | 0.304348 |
| - | hypothetical protein, DUF1456 family |  |  | 0.321267 |
| - | hypothetical protein |  |  | 0.322581 |
| ychF | ATPase, K+-dependent, ribosome-associated |  |  | 0.324561 |
| rsd | stationary phase protein |  |  | 0.328571 |
| - | - |  |  | 0.338938 |
| cycA | D-alanine/D-serine/glycine transporter |  |  | 0.340426 |
| msgA | virulence protein MsgA |  |  | 0.35 |
| udk | uridine/cytidine kinase |  |  | 0.355482 |
| gltP | glutamate/aspartate:proton symporter |  |  | 0.359375 |
| - | putative iron transporter |  |  | 0.3625 |
| rsmC | 16S rRNA m(2)G1207 methyltransferase |  |  | 0.362637 |
| emrD | multidrug efflux system protein |  |  | 0.363636 |
| trmA | tRNA m(5)U54 methyltransferase, SAM-dependen |  |  | 0.367647 |
| arnC | undecaprenyl phosphate-L-Ara4FN transferase |  |  | 0.368601 |
| rluB | 23S rRNA pseudouridylate synthase |  |  | 0.371069 |
| trmJ | tRNA mC32/mU32 methyltransferase, SAM-dependent |  |  | 0.372549 |
| betI | DNA-binding transcriptional repressor |  |  | 0.375 |
| lpxA | UDP-N-acetylglucosamine acetyltransferase |  |  | 0.379233 |
| - | antisense: rplM |  |  | 0.380414 |
| bioF | 8-amino-7-oxononanoate synthase |  |  | 0.384615 |
| rhlE | ATP-dependent RNA helicase |  |  | 0.3861 |
| - | antisense: pgk |  |  | 2.469325 |
| dcuC | anaerobic C4-dicarboxylate transport |  |  | 2.506024 |
| pepT | peptidase T |  |  | 2.634409 |
| gntT | gluconate transporter, high-affinity GNT I system |  |  | 2.879518 |
| torC | trimethylamine N-oxide (TMAO) reductase I, cytochrome c-type subunit |  |  | 3.111111 |
| manX | mannose-specific PTS system IIA component |  |  | 3.571429 |
| - | short-chain dehydrogenase/reductase |  |  | 3.678161 |
| yfbT | putative phosphatase |  |  | 3.872727 |
| ybhI | putative transporter |  |  | 4.135135 |
| - | hypothetical protein |  |  | 4.165432 |
| - | hypothetical protein |  |  | 4.34375 |
| uspG | universal stress protein UP12 |  |  | 4.888889 |
| - | hypothetical protein |  |  | 6.7 |
| edd | 6-phosphogluconate dehydratase |  |  | 6.8 |
| cadB | putative lysine/cadaverine transporter |  |  | 6.878788 |
| frdA | fumarate reductase, catalytic and NAD/flavoprotein subunit |  |  | 7.6 |
| menB | dihydroxynaphthoic acid synthetase |  |  | 7.73913 |
| - | putative transcriptional regulator |  |  | 7.777778 |
| nirD | nitrite reductase, NAD(P)H-binding, small subunit |  |  | 7.974359 |
| - | antisense: cadB |  |  | 8.147059 |
| menE | o-succinylbenzoate-CoA ligase |  |  | 8.166667 |
| frdB | fumarate reductase, Fe-S subunit |  |  | 8.425532 |
| galT | galactose-1-phosphate uridylyltransferase |  |  | 9.833333 |
| - | antisense: SMWW4_v1c38880 |  |  | 10.15909 |
| menC | o-succinylbenzoyl-CoA synthase |  |  | 10.66667 |
| - | lysine decarboxylase |  |  | 11.72986 |
| - | transcriptional regulator |  |  | 14 |
| frdC | fumarate reductase, membrane anchor subunit |  |  | 14.78571 |
| - | 2-dehydro-3-deoxyphosphogluconate aldolase |  |  | 15.5 |
| - | nitrite transporter |  |  | 19 |
| cysG | siroheme synthase |  |  | 19.33333 |
| - | hypothetical protein |  |  | 22 |
| - | - |  |  | 28.13793 |
| - | nitrate reductase, alpha subunit |  |  | 33.83333 |
| yhjX | Inner membrane protein, putative oxalate-formate antiporter |  |  | 67.66667 |
| - | nitrate reductase, beta subunit |  |  | 81 |
